# Hypoxia inducible factor 1α in vascular smooth muscle cells promotes angiotensin II-induced vascular remodeling via activation of CCL7-mediated macrophage recruitment

**DOI:** 10.1038/s41419-019-1757-0

**Published:** 2019-07-18

**Authors:** Dan Qi, Ming Wei, Shiyu Jiao, Yanting Song, Xia Wang, Guomin Xie, Joseph Taranto, Ye Liu, Yan Duan, Baoqi Yu, Huihua Li, Yatrik M. Shah, Qingbo Xu, Jie Du, Frank J. Gonzalez, Aijuan Qu

**Affiliations:** 1 Department of Physiology and Pathophysiology, School of Basic Medical Sciences, Capital Medical University; Key Laboratory of Remodeling-Related Cardiovascular Diseases, Ministry of Education, Beijing, China; 2 Molecular & Integrative Physiology, Internal Medicine Division of Gastroenterology, University of Michigan School of Public Health, Ann Arbor, MI USA; 3 Internal Medicine Division of Gastroenterology, University of Michigan School of Public Health, Ann Arbor, MI USA; 4 Rogel Cancer Center, University of Michigan Medical School, Ann Arbor, MI USA; 5 Department of Nutrition and Food Hygiene, School of Public Health, Department of Cardiology, Institute of Cardiovascular Diseases, First Affiliated Hospital of Dalian Medical University, Dalian, China; 6 School of Cardiovascular Medicine and Sciences, King’s College of London, London, UK; 7 Beijing Anzhen Hospital of Capital Medical University and Beijing Institute of Heart, Lung and Blood Vessel Diseases, Beijing, China; 8 Laboratory of Metabolism, Center for Cancer Research, National Cancer Institute, National Institutes of Health, Bethesda, MD USA

**Keywords:** Vascular diseases, Inflammation

## Abstract

The process of vascular remodeling is associated with increased hypoxia. However, the contribution of hypoxia-inducible factor 1α (HIF1α), the key transcription factor mediating cellular hypoxic responses, to vascular remodeling is established, but not completely understood. In the angiotensin II (Ang II)-induced vascular remodeling model, HIF1α was increased and activated in vascular smooth muscle cells (VSMCs). Selective genetic disruption of *Hif1a* in VSMCs markedly ameliorated Ang II-induced vascular remodeling, as revealed by decreased blood pressure, aortic thickness, collagen deposition, inflammation, and aortic stiffness. VSMC *Hif1a* deficiency also specifically suppressed Ang II-induced infiltration of CD45^+^CD11b^+^F4/80^+^CD206^−^ M1 macrophages into the vessel. Mechanistically, HIF1α deficiency in VSMCs dramatically suppressed the expression of CCL7, a chemokine critical for macrophage recruitment. Bioinformatic analysis and chromatin immunoprecipitation assays revealed three functional hypoxia-response elements in the *Ccl7* promoter, indicating that *Ccl7* is a direct HIF1α target gene. Blocking CCL7 with antibody in vivo alleviated Ang II-induced hypertension and vascular remodeling, coincident with decreased macrophage infiltration. This study provides direct evidence that HIF1α activation in VSMCs exacerbates Ang II-induced macrophage infiltration and resultant vascular remodeling via its target gene *Ccl7*, and thus may serve as a potential therapeutic target for remodeling-related vascular disease.

## Introduction

Hypertension is the most common preventable risk factor for cardiovascular disease and the leading single contributor to mortality and disability worldwide^1^. Despite its prevalence, the pathogenesis of essential hypertension remains poorly defined. It has become increasingly evident that vascular remodeling is the pathological basis for hypertension, of which, inflammation plays a critical role^2^. The transmigrated and accumulated innate and adaptive immune cells, such as monocytes/ macrophages, T cells, and dendritic cells, in the vasculature system release cytokines, interact with vascular smooth muscle cells (VSMCs), promote oxidative stress, and finally result in vascular remodeling and hypertension^3^. However, little is known about how these immune cells are mobilized and recruited into interstitium of affected arteries.

Previous results revealed that angiotensin II (Ang II), norepinephrine, and mechanical strain could increase monocyte chemoattractant protein 1 (MCP-1) expression^4^. Increased expression of specific chemokines within injured tissues directs the diapedesis of mononuclear cells across the vascular endothelium into the tissue parenchyma^5^. Blocking the MCP-1/CCR-2 axis and other CC chemokine pathways (CCR1, CCR5) prevented Ang II-induced monocyte adhesion to the microvasculature^6^. In the context of hypertension, identifying the chemokines that drive the infiltration of immune cells into the vascular require further investigation.

Hypoxia-inducible factor 1 (HIF1) is a key transcription factor controlling the cellular responses to hypoxia^7^. HIF1 is a heterodimer consisting of 1α and 1β subunits, of which, 1α is oxygen sensitive while 1β is constitutively expressed^8,9^. Under normoxia, HIF1α is hydroxylated by prolyl hydroxylases (PHD), recognized by the E3 ubiquitin-ligase von Hippel Lindau (VHL) and subsequently degraded by the ubiquitin–proteasomal system. Upon hypoxia, HIF1α protein is stabilized and translocated to the nucleus, where it dimerizes with HIF1β, and binds to the hypoxic response elements (HREs) to regulate target gene transcription^10,11^. Several hundred of direct HIF1 target genes have been identified and many encode proteins critical for glycolysis, apoptosis, inflammation, and angiogenesis^12,13^. Recent studies suggest that HIF1α may also be involved in cell proliferation, migration, differentiation, and extracellular matrix metabolism, which are key pathologies associated with vascular remodeling-related diseases^14,15^. For example, HIF1α deficiency in SMCs caused decreased vascular inflammation and atherosclerosis in the atherosclerotic-prone *Apoe*^−/−^ mice^16^. HIF1α disruption in myeloid cells inhibited neointimal formation in wire-injured femoral arteries in mice^17^. HIF1α deficiency in T cells led to prominent neointimal hyperplasia in external vascular polyethylene cuff-induced femoral injury model^18^. Systemic HIF1 inhibition decreases carotid artery post-injury remodeling in rats^19^. However, the role of VSMC HIF1α in Ang II-induced vascular remodeling has not been sufficiently explored^20,21^.

To elucidate the role of HIF1α in VSMCs during Ang II-induced vascular remodeling, mice lacking HIF1α expression in VSMCs were generated. The present study revealed that HIF1α activation in VSMCs promotes its target gene *Ccl7* expression leading to increased macrophage recruitment and consequently exacerbated vascular remodeling in Ang II-induced hypertensive process, suggesting that HIF1α and its downstream CCL7 may serve as potential targets for hypertensive disease.

## Results

### HIF1α is activated in VSMCs during Ang II-induced vascular remodeling

**Fig. 1:**
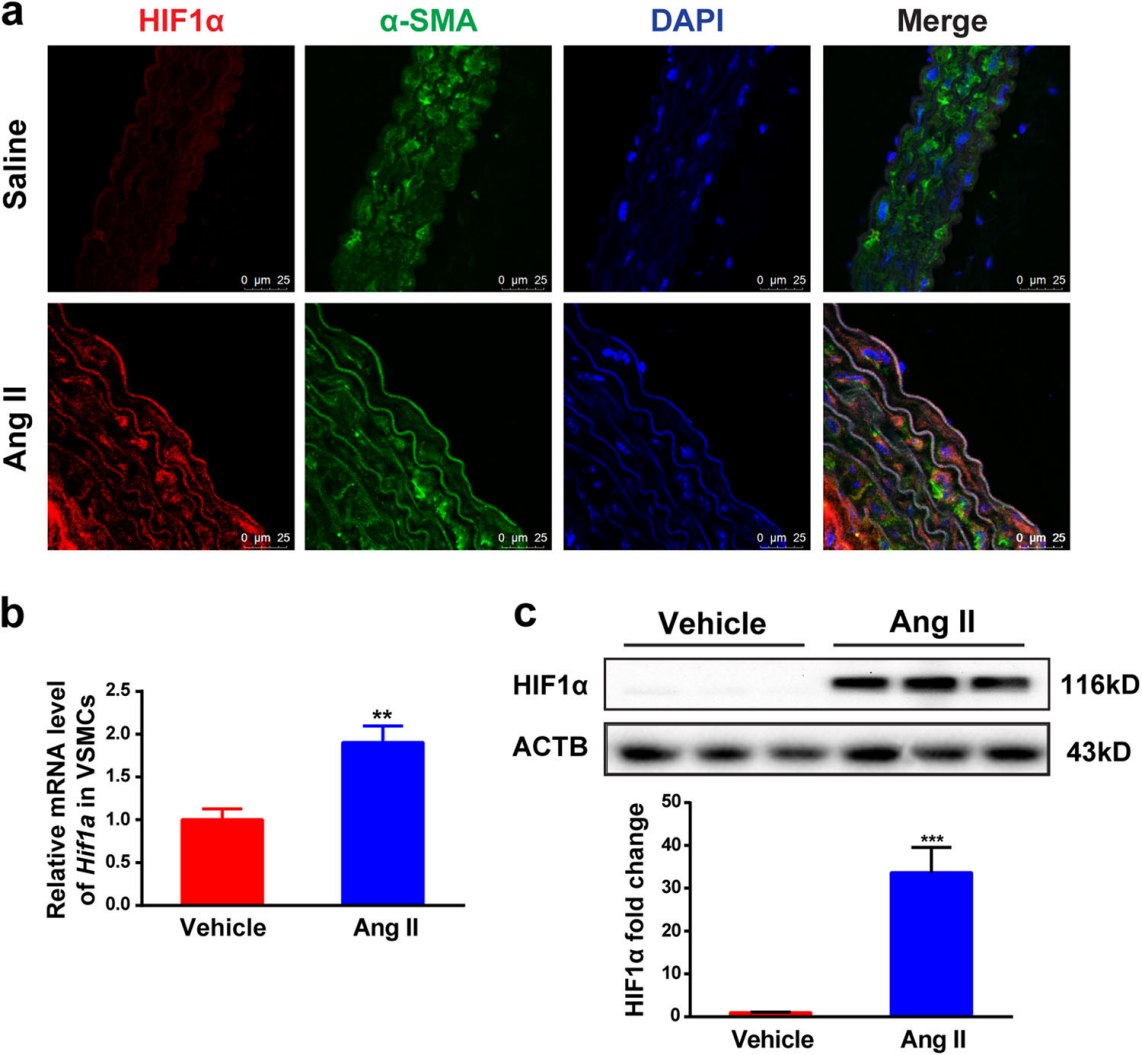
HIF1α is activated in VSMCs during Ang II-induced vascular remodeling. WT mice were infused with saline or 1000 ng/kg/min Ang II for 28 days. **a** Immunofluorescence analysis of representative cross-sections of mice aortas for HIF1α (red) and α-SMA (green), nuclei was stained with DAPI. VSMCs were isolated form WT mice and treated with 1 μM Ang II for 24 h. **b** *Hif1a* mRNA was measured by qPCR analysis. **c** HIF1α protein was detected by western blot. **P < 0.01, ***P < 0.001, *n* = 3 per group, statistical significance was determined by the unpaired *t-*test

To determine the role of HIF1α in VSMCs during Ang II-induced vascular remodeling, 10-week-old male wild-type (WT) mice were infused with saline or 1000 ng/kg/min Ang II for 28 days to establish the Ang II-induced vascular remodeling model. Immunofluorescent staining demonstrated that HIF1α colocalized with DAPI in α-SMA-marked VSMCs in the Ang II-treated vessels, but not in the saline-treated group (Fig. 1a). To further confirm whether HIF1α could be activated in VSMCs during Ang II-induced vascular remodeling, primary VSMCs isolated from C57BL/6J mice were treated with Ang II for 24 h. *Hif1a* mRNA was increased upon Ang II treatment and this was coincident with a significant increase of HIF1α protein upon Ang II stimulation (Fig. 1b, c), consistent with previous studies^22,23^. These results suggest that HIF1α is activated in VSMCs during Ang II-induced vascular remodeling.

### HIF1α deficiency in SMCs suppresses Ang II-induced vascular remodeling in mice

To investigate the role of HIF1α in VSMCs in Ang II-induced vascular remodeling, *Hif1a*^fl/fl^ mice^24^ were crossed with SM22alpha-Cre mice^25^ to generate VSMC-specific *Hif1a*-deficient mice (Supplementary Fig. 2a). *Hif1a* mRNA level was reduced approximately 90% in VSMCs and 70% in aortic tissues from *Hif1a*^ΔSMC^ mice (Supplementary Fig. 2b). As HIF1α protein is undetectable under normoxia, primary VSMCs isolated from *Hif1a*^fl/fl^ and *Hif1a*^ΔSMC^ mice were treated with the hypoxia mimic CoCl_2_. HIF1α protein was dramatically decreased in CoCl_2_-treated VSMCs from *Hif1a*^ΔSMC^ mice (Supplementary Fig. 2c, d), suggesting successful construction of VSMC-specific *Hif1a*-deficient mice.

**Fig. 2:**
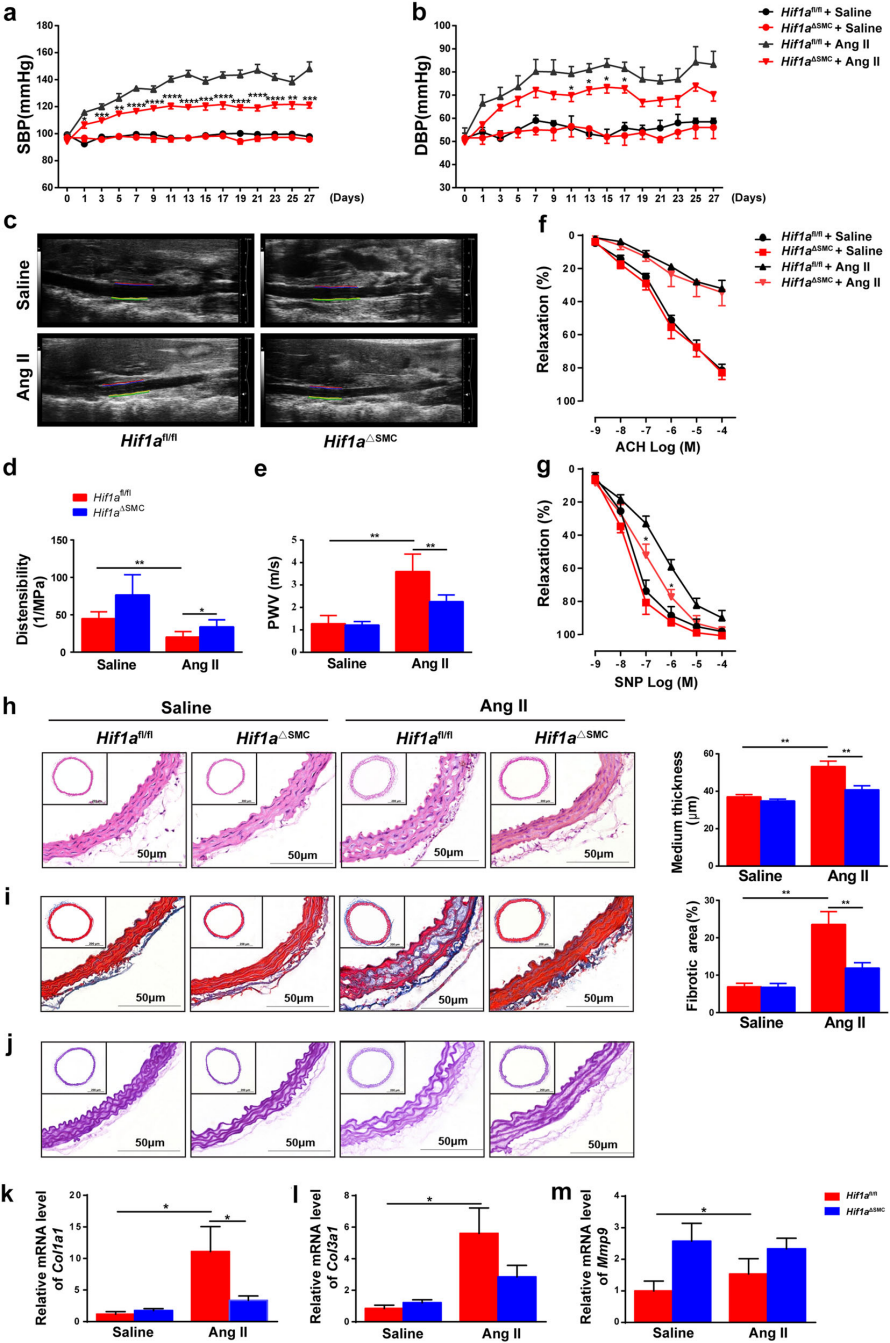
HIF1α deficiency in SMCs suppresses Ang II-induced vascular remodeling in mice. *Hif1a*^fl/fl^ and *Hif1a*^ΔSMC^ mice were infused with saline or Ang II (1000 ng/kg/min) for 28 days. **a** SBP and **b** DBP were measured by the tail-cuff method. *P < 0.05, **P < 0.01, ***P < 0.001 vs. *Hif1a*^fl/fl^ + Ang II; *n* = 10 per group, statistical significance was determined by two-way ANOVA analysis. M-mode ultrasound of abdominal aorta was acquired **c**, and the distensibility (**d**) as well as pulse wave velocity (PWV) (**e**) were measured. *P < 0.05, **P < 0.01, *n* = 6 per group. **f**, **g** Concentration–response curves of endothelium-dependent (acetylcholine, Ach) and endothelium-independent (sodium nitroprusside, SNP) relaxation. *P < 0.05 vs. *Hif1a*^fl/fl^ + Ang II, *n* = 6 per group. **h** Representative images of H&E staining and the mean medium thickness for the aortas. **i** Representative images of Masson’s trichrome staining and the fibrotic area of each group were analyzed. **j** Representative images of Elastin staining for the aortas. *P < 0.05, **P < 0.01, *n* = 8/saline group, *n* = 12/Ang II group. **k–m** Aortic *Col1a1, Col3a1*, and *Mmp9* mRNAs were measured by qPCR. *P < 0.05, *n* = 6 per group. Statistical significance was determined by one-way ANOVA test followed by the unpaired *t*-test

Without challenge, no difference in basal systolic (SBP) or diastolic pressure (DBP) was found between *Hif1a*^fl/fl^ and *Hif1a*^ΔSMC^ mice. When infused with Ang II, *Hif1a*^fl/fl^ mice exhibited a significant elevation of SBP and DBP, whereas this phenomenon was dramatically blunted in *Hif1a*^ΔSMC^ mice (Fig. 2a, b). Impaired elasticity and resultant stiffening are characteristic of vascular remodeling^24^. As determined by vascular ultrasound, Ang II induced a significant decrease of distensibility and increase of PWV in the aortas from *Hif1a*^fl/fl^ mice, whereas this effect was reversed by *Hif1a* disruption in VSMCs (Fig. 2c–e), indicating improved vessel elasticity in *Hif1a*^ΔSMC^ mice. To examine the mechanism by which HIF1α deficiency in VSMCs ameliorated vessel elasticity, ex vivo vascular function in Ang II-infused *Hif1a*^fl/fl^ and *Hif1a*^ΔSMC^ mice was measured. Intact aortas were isolated from *Hif1a*^fl/fl^ and *Hif1a*^ΔSMC^ mice after saline or Ang II infusion, and concentration–relaxation curves in response to acetylcholine (Ach) or sodium nitroprusside (SNP) were examined. Consistent with previous observations^25^, chronic Ang II infusion dramatically impaired endothelium-dependent vasodilatation to Ach compared with saline-treated control, although no differences between *Hif1a*^fl/fl^ and *Hif1a*^ΔSMC^ mice was observed. However, endothelium-independent vasodilatation to SNP was improved in *Hif1a*^ΔSMC^ mice (Fig. 2f, g). These results indicate that HIF1α deficiency in VSMCs prevented Ang II-induced VSMC dysfunction.

Furthermore, hematoxylin and eosin (H&E) and Masson’s trichrome staining revealed a significant reduction of medial thickening as well as vascular fibrosis in *Hif1a*^ΔSMC^ mice compared with *Hif1a*^fl/fl^ mice (Fig. 2h, i). Elastin staining with the Gomori’s aldehyde-fuchsin staining demonstrated disordered elastic lamina in Ang II-infused *Hif1a*^fl/fl^ mice, which was reversed by HIF1α disruption in VSMCs (Fig. 2j). Consistently, the *Col1a1 and Col3a1* mRNAs, encoding typical fibrotic markers, were suppressed in *Hif1a*^ΔSMC^ mice compared with *Hif1a*^fl/fl^ mice (Fig. 2k–m). These data indicate that HIF1α disruption in VSMCs improves Ang II-induced vascular remodeling.

Ang II also increased *Hif2α* mRNA and HIF2α protein in aortas, but there were no differences in the extent of increase between *Hif1α*^fl/fl^ and *Hif1a*^ΔSMC^ mice (Supplementary Fig. 3a, b). In vitro, the Ang II induced HIF2α protein expression was not affected in HIF1α-deficient VSMCs (Supplementary Fig. 3c).

### HIF1α deficiency in VSMCs inhibits Ang II-induced macrophage infiltration and vascular inflammation

**Fig. 3:**
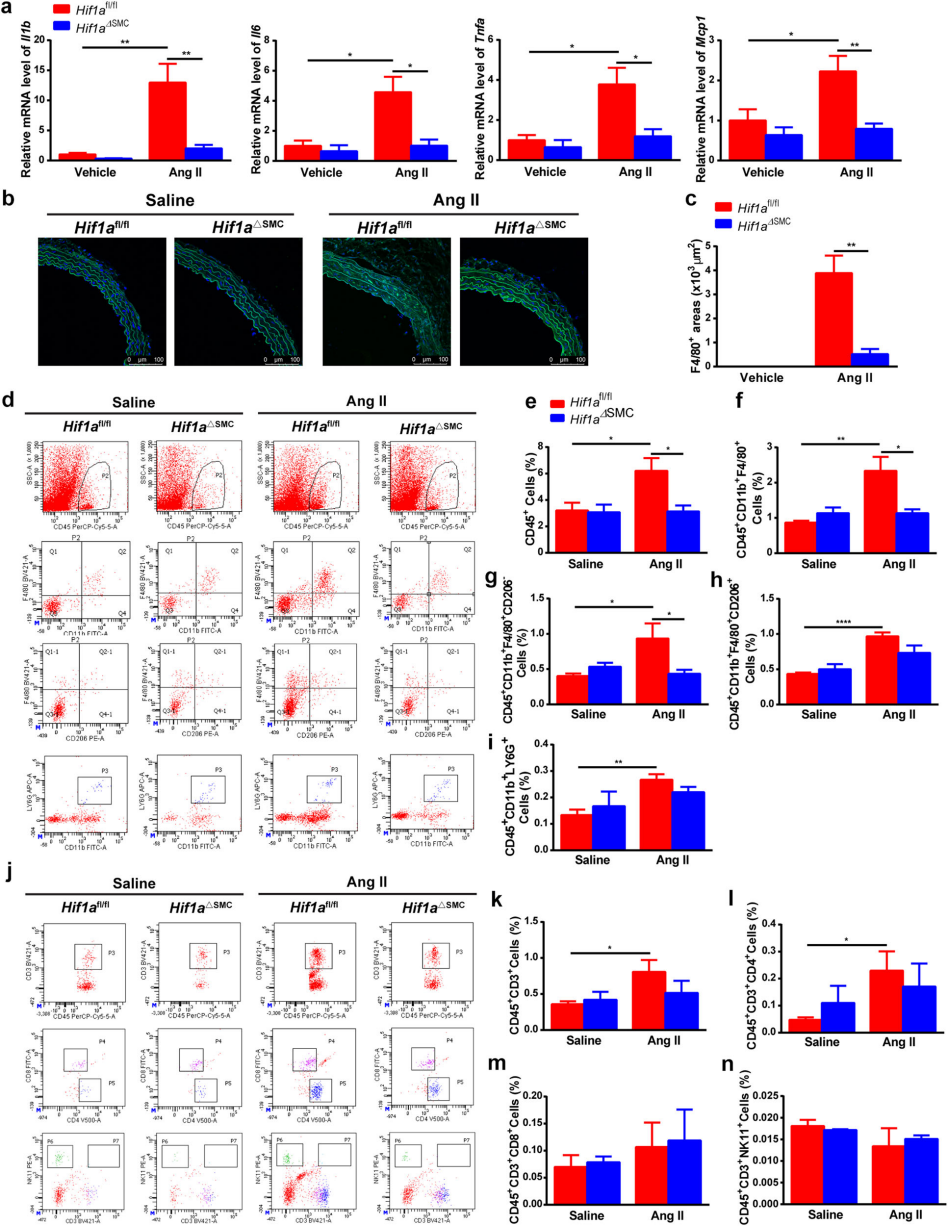
SMC-specific HIF1α deficiency abolishes Ang II-induced M1 macrophage infiltration and vascular inflammation. *Hif1a*^fl/fl^ and *Hif1a*^ΔSMC^ mice were infused with saline or 1000 ng/kg/min Ang II for 28 days. **a** *Il1b*, *Il6*, *Tnfa*, *Mcp1* mRNAs in aortas after saline or angiotensin II infusion for 28 days were measured by qPCR. *P < 0.05, **P < 0.01, *n* = 6 per group. **b** Immunofluorescence staining of representative cross-sections of mice aortas for the F4/80-positive (green) cells and quantification (**c**) *P *<* 0.05, *n* = 6, statistical significance was determined by the unpaired *t*-test. Flow cytometry analysis was performed for the aortas (**d**, **j**) and CD45^+^ cells (**e)**, CD45^+^CD11b^+^F4/80^+^ macrophages (**f**), CD45^+^CD11b^+^F4/80^+^CD206^−^ M1 macrophages (**g**), CD45^+^CD11b^+^F4/80^+^CD206^+^ M2 macrophages (**h**), CD45^+^CD11b^+^LY6G^+^ neutrophils (**i)**, CD45^+^CD3^+^ T cells (**k**), CD45^+^CD3^+^CD4^+^ T cells (**l**),CD45^+^CD3^+^CD8^+^ T cells (**m**), and CD45^+^CD3^+^NK11^+^ NKT cells (**n**) were quantified, respectively. *P < 0.05, **P < 0.01, ***P < 0.001, *n* = 6 per group. Statistical significance was determined by one-way ANOVA test followed by the unpaired *t*-test

Immune cell infiltration and inflammation play an important role in Ang II-induced vascular remodeling^26^. Ang II induced significant increases of the proinflammatory *Il1b*, *Il6*, *Tnfa*, and *Mcp1* mRNAs in *Hif1a*^fl/fl^ mice, which were completely abolished in *Hif1a*^ΔSMC^ mice (Fig. 3a). Immunofluorescence staining showed that Ang II-caused F4/80^+^ macrophage infiltration in *Hif1a*^fl/fl^ mice was dramatically blunted in *Hif1a*^ΔSMC^ mice (Fig. 3b, c). To further elucidate which type of immune cells was involved in this process, flow cytometry assays were performed with aortas (Fig. 3d). Ang II evoked infiltration of CD45^+^ myelomonocytes (Fig. 3e), especially F4/80^+^ macrophages (Fig. 3f) into the aorta; however, this effect was diminished in *Hif1a*^ΔSMC^ mice. Interestingly, the CD45^+^CD11b^+^F4/80^+^CD206^−^ M1 (Fig. 3g), but not CD45^+^CD11b^+^F4/80^+^CD206^+^ M2 macrophages (Fig. 3h), neutrophils (Fig. 3i), or T cells (Fig. 3j–n), were markedly decreased by HIF1α disruption in VSMCs. These results suggest that HIF1α deficiency in VSMCs may specifically suppress Ang II-induced M1 macrophage infiltration and vascular inflammation.

### HIF1α deficiency leading to low CCL7 expression suppresses macrophage recruitment by Ang II-induced VSMC

**Fig. 4:**
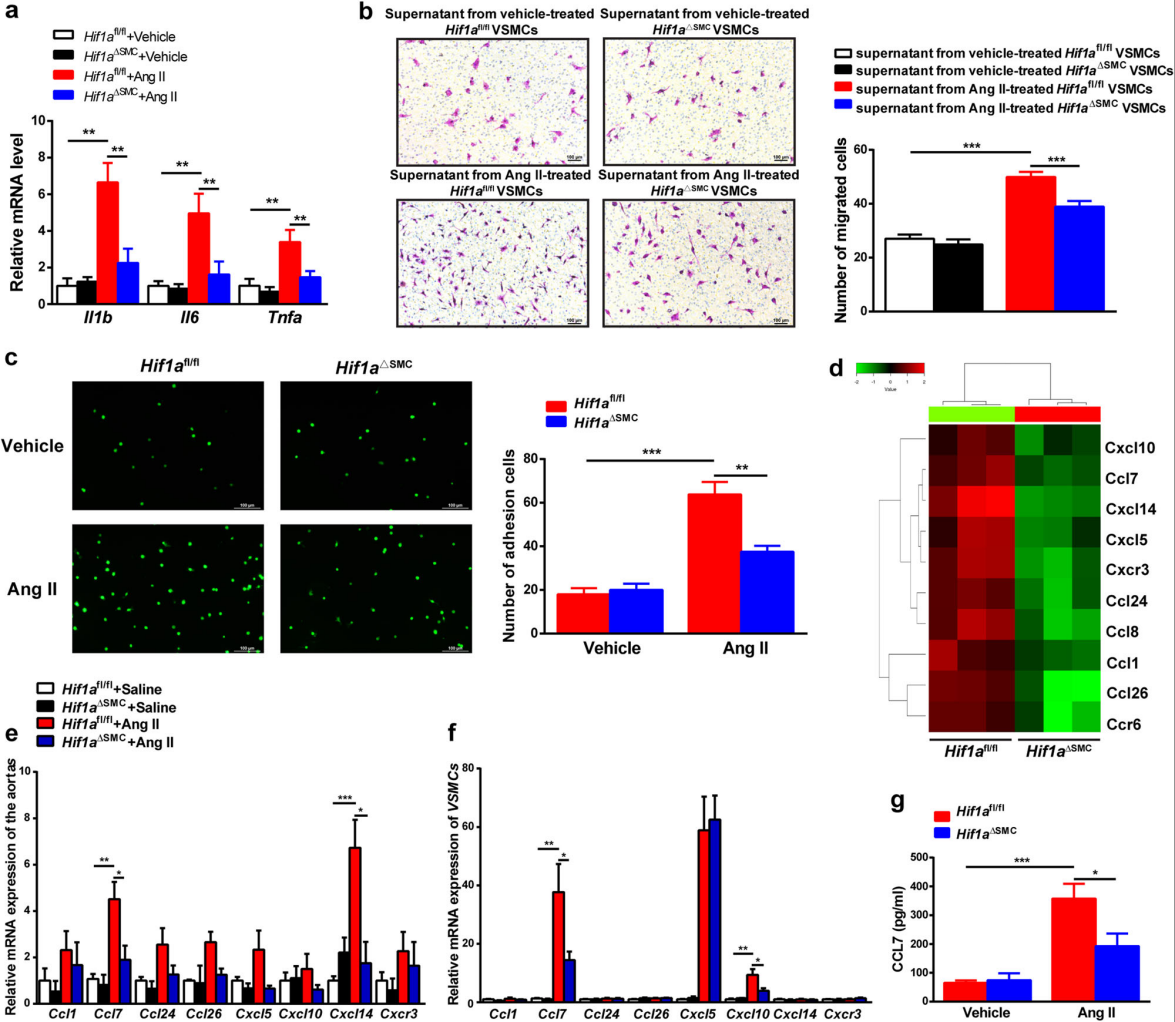
HIF1α deficiency leading to low CCL7 expression suppresses macrophage recruitment by Ang II-induced VSMCs. **a** *Il1b, Il6*, and *Tnfa* mRNA levels in 1 μM Ang II-treated *Hif1a*^fl/fl^ and *Hif1a*^ΔSMC^ VSMCs. **P < 0.01, *n* = 6 (independent experiments) per group. **b** Macrophage chemotaxis driven by supernatants from vehicle or 1 μM Ang II-treated *Hif1a*^fl/fl^ and *Hif1a*^ΔSMC^ VSMCs. ***P < 0.001, *n* = 6 (independent experiments) per group. **c** Adhesion assay of Calcein-AM-labeled macrophages with vehicle or 1 μM Ang II-treated *Hif1a*^fl/fl^ and *Hif1a*^ΔSMC^ VSMCs. **P < 0.01, ***P < 0.001, *n* = 6 (independent experiments) per group. **d** The cluster heat map of expression values for differentially expressed chemokines in the VSMCs after 150 μM CoCl_2_ treatment. Validation of chemokine mRNAs by qPCR in Ang II-treated aortas (**e**) and VSMCs (**f**) *P < 0.05, **P < 0.01, ***P < 0.001, *n* = 6 (independent experiments) per group, statistical significance was determined by one-way ANOVA test followed by the unpaired *t*-test. **g** Detection of CCL7 levels by ELISA in the supernatants from vehicle or Ang II-treated VSMCs. *P < 0.05, ***P < 0.001, *n* = 6 (independent experiments) per group

To clarify the role of HIF1α in VSMC function in vitro, VSMCs from *Hif1a*^fl/fl^ mice and *Hif1a*^ΔSMC^ mice were treated with Ang II. *Hif1a* deficiency abolished Ang II-evoked inflammatory gene expression, such as *Il1b*, *Il6*, and *Tnfa* in VSMCs (Fig. 4a). As decreased M1 macrophage infiltration was observed in Ang II-infused *Hif1a*^ΔSMC^ aortas compared with *Hif1a*^fl/fl^ aortas, the interaction between macrophages and VSMCs was evaluated. The supernatant from Ang II-treated *Hif1a*^fl/fl^ VSMCs significantly increased macrophage migration, whereas this effect was markedly blunted by *Hif1a* disruption in VSMCs (Fig. 4b). Moreover, the adhesion between macrophages and Ang II-treated VSMCs was also suppressed by VSMC *Hif1a* deficiency (Fig. 4c).

Chemokines mediate the recruitment of macrophages toward injured vessels^27^. To explore which chemokines increased upon HIF1α activation in VSMCs, microarray analysis was performed in *Hif1a*^fl/fl^ and *Hif1a*^ΔSMC^ VSMCs treated with CoCl_2_. The majority of downregulated mRNAs in *Hif1a*^ΔSMC^ VSMCs were chemokines, such as *Ccl1, Ccl7, Ccl24, Ccl26, Cxcl5, Cxcl10, Cxcl14*, and *Cxcr3* (Fig. 4d). However, when validated in Ang II-treated aortas and VSMCs, *Ccl7* was the only chemokine obviously changed both in aortas (Fig. 4e) and in VSMCs (Fig. 4f). Furthermore, Ang II markedly increased CCL7 secretion from *Hif1a*^fl/fl^ VSMCs, which was reversed by *Hif1a* disruption (Fig. 4g). These data suggest that CCL7 might be a key chemokine mediating the effects of HIF1α activation in VSMCs.

### *Ccl7* is a novel HIF1α but not HIF2α direct target gene in VSMCs

**Fig. 5:**
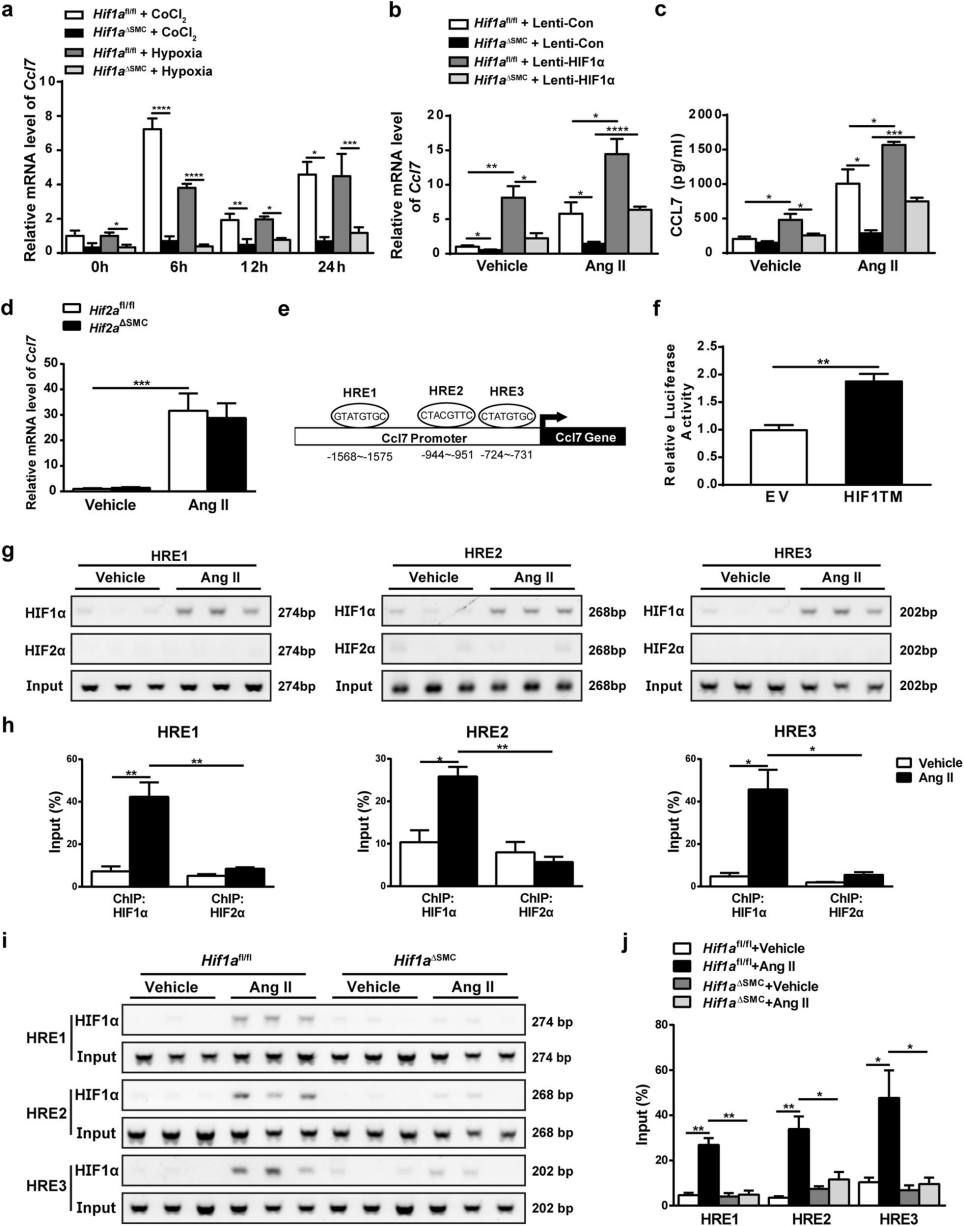
*Ccl7* is a HIF1α direct target gene. **a** qPCR analysis of *Ccl7* mRNA expression in *Hif1a*^fl/fl^ and *Hif1a*^ΔSMC^ VSMCs treated with vehicle, CoCl_2_, normoxia, or hypoxia (2% O_2_) for 6, 12, and 24 h. *P < 0.05, **P < 0.01, ***P < 0.001, *n* = 6 (independent experiments) per group. VSMCs isolated from *Hif1a*^fl/fl^ mice and *Hif1a*^ΔSMC^ mice were infected with oxygen-stable HIF1α-expressing lentivirus, and then treated with Ang II for 24 h, **b** *Ccl7* mRNA was measured by qPCR and **c** CCL7 protein was detected by ELISA. **d** qPCR analysis of *Ccl7* mRNA expression in vehicle or Ang II-treated *Hif2a*^fl/fl^ and *Hif2a*^ΔSMC^ VSMCs. **e** Schematic diagram of the mouse *Ccl7* promoter illustrating the HREs in the regulatory region; the upstream regions were numbered in relation to the transcription initiation site. **f** Luciferase-reporter constructs under the control of the mouse *Ccl7* promoter. HEK293T human embryonic kidney cells transiently transfected with the luciferase construct, and cotransfected with empty vector or HIF1a expression plasmids. Standard dual-luciferase assays were performed. EV, empty vector. **P < 0.01, *n* = 3. **g**, **h** ChIP assays of vehicle or Ang II-treated wild-type VSMCs using HIF1α or HIF2α antibodies. Data were normalized to input. *P < 0.05, **P < 0.01, *n* = 6 per group. **i**, **j** ChIP assays of vehicle or Ang II-treated *Hif1a*^fl/fl^ and *Hif1a*^ΔSMC^ VSMCs using HIF1α antibody. Data were normalized to input. *P < 0.05, **P < 0.01, *n* = 6 per group. Statistical significance was determined by one-way ANOVA test followed by the unpaired *t*-test

To identify whether *Ccl7* is a HIF1α target gene, VSMCs from *Hif1a*^fl/fl^ and *Hif1a*^ΔSMC^ mice were treated with hypoxia or CoCl_2_ for 6, 12, 24 h and *Ccl7* mRNA measured. CoCl_2_ or hypoxia significantly induced *Ccl7* mRNA expression in *Hif1a*^fl/fl^ VSMCs, but this effect was completely abolished in *Hif1a*-deficient VSMCs (Fig. 5a). To further confirm the involvement of HIF1α in regulating CCL7 expression, forced overexpression of oxygen-stable HIF1α by lentivirus was carried out in VSMCs from *Hif1a*^fl/fl^ and *Hif1a*^ΔSMC^ mice (Supplementary Fig. 4). Forced HIF1α overexpression significantly increased *Ccl7* mRNA (Fig. 5b) and CCL7 protein levels (Fig. 5c) in *Hif1a*^fl/fl^ VSMCs with or without Ang II treatment. More interestingly, forced overexpression of HIF1α dramatically reversed the suppressed *Ccl7* mRNA (Fig. 5b) and CCL7 protein levels (Fig. 5c) in *Hif1a*^ΔSMC^ VSMCs upon Ang II administration, indicating that HIF1α is critical for Ang II-induced CCL7 expression in VSMCs.

Both HIF1 α and HIF2α can be activated under hypoxia. To investigate whether HIF2α could also regulate CCL7 expression, VSMCs from *Hif2a*^fl/fl^ and *Hif2a*^ΔSMC^ mice were treated with Ang II and *Ccl7* mRNA was quantified. However, *Hif2a* deficiency in VSMCs had no effect on Ang II-induced *Ccl7* mRNA expression (Fig. 5d). Bioinformatic analysis revealed three putative HIF-response elements (HRE) in the *Ccl7* promoter (Fig. 5e). To assess whether *Ccl7* could be a novel direct target of HIF1α, *Ccl7*-promoter luciferase assays were performed. Cotransfection with an HIF1α expression plasmid strongly increased luciferase expression (Fig. 5f). Chromatin-immunoprecipitation (ChIP) assays were then performed to identify the functional HREs in the *Ccl7* promoter. Primers flanking three HREs specifically amplified the DNA sequence immunoprecipitated by the HIF1α but not HIF2α antibody in Ang II-treated VSMCs (Fig. 5g, h). Next, to further confirm the specificity for the binding of HIF1α to HREs on *Ccl7* promoter, ChIP assays were also performed in *Hif1a*^ΔSMC^ VSMCs. No obvious amplification of the three HREs was obtained from Ang II-treated *Hif1a*-deficient VSMCs (Fig. 5i, j). These data demonstrated that *Ccl7* is a HIF1α but not HIF2α direct target gene in VSMCs.

### CCL7 neutralization suppresses Ang II-induced macrophage migration and vascular remodeling in mice

**Fig. 6:**
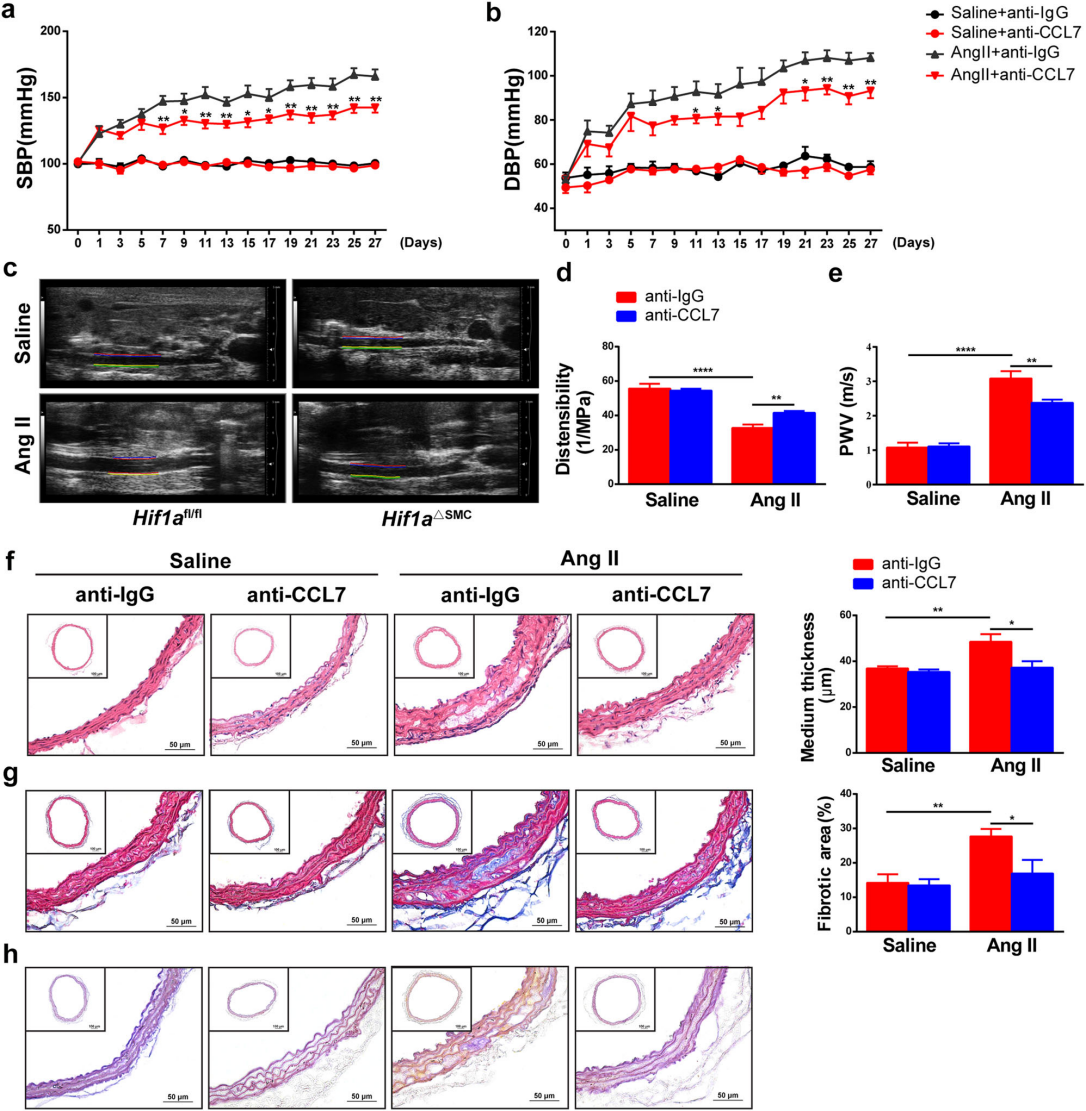
CCL7 neutralization suppresses Ang II-induced vascular remodeling. Wild-type mice were infused with saline or 1000 ng/kg/min Ang II for 28 days in the presence of normal IgG (anti-IgG) or CCL7-neutralizing antibody (anti-CCL7). SBP (**a**) and DBP (**b**) were measured by the tail-cuff method. *P < 0.05, **P < 0.01 vs. Ang II + anti-IgG; *n* = 8 per group, statistical significance was determined by two-way ANOVA test. **c** M-mode ultrasound of abdominal aortas, **d** measurement of distensibility, and **e** pulse wave velocity (PWV). *P < 0.05, **P < 0.01, *n* = 8 per group. **f** H&E staining of arterial sections and the medium thickness were measured. **g** Masson’s trichrome staining of arterial sections and the fibrotic area were measured. **h** Elastin staining was performed with the Gomori’s aldehyde-fuchsin of arterial sections. *P < 0.05, **P < 0.01, *n* = 8 per group. Statistical significance was determined by one-way ANOVA test followed by the unpaired *t*-test

To assess the importance of CCL7 in Ang II-induced vascular remodeling, control or CCL7-neutralizing antibodies at a dose of 2 μg/mouse were intravenously administered every other day from 1 day before Ang II infusion. CCL7 blockade by neutralizing antibody greatly attenuated Ang II-induced SBP (Fig. 6a) and DBP elevation (Fig. 6b). As indicated by increased distensibility (Fig. 6c, d) and decreased PWV (Fig. 6e) compared with normal IgG-treated aortas after Ang II infusion, neutralizing CCL7 greatly improved vessel elasticity in vivo. Morphologically, neutralizing CCL7 markedly alleviated Ang II-induced medial thickness (Fig. 6f), collagen deposition (Fig. 6g), and disordered elastic lamina (Fig., 6h) in aortic tissues, suggesting that CCL7 neutralization improved Ang II-induced vascular remodeling.

**Fig. 7:**
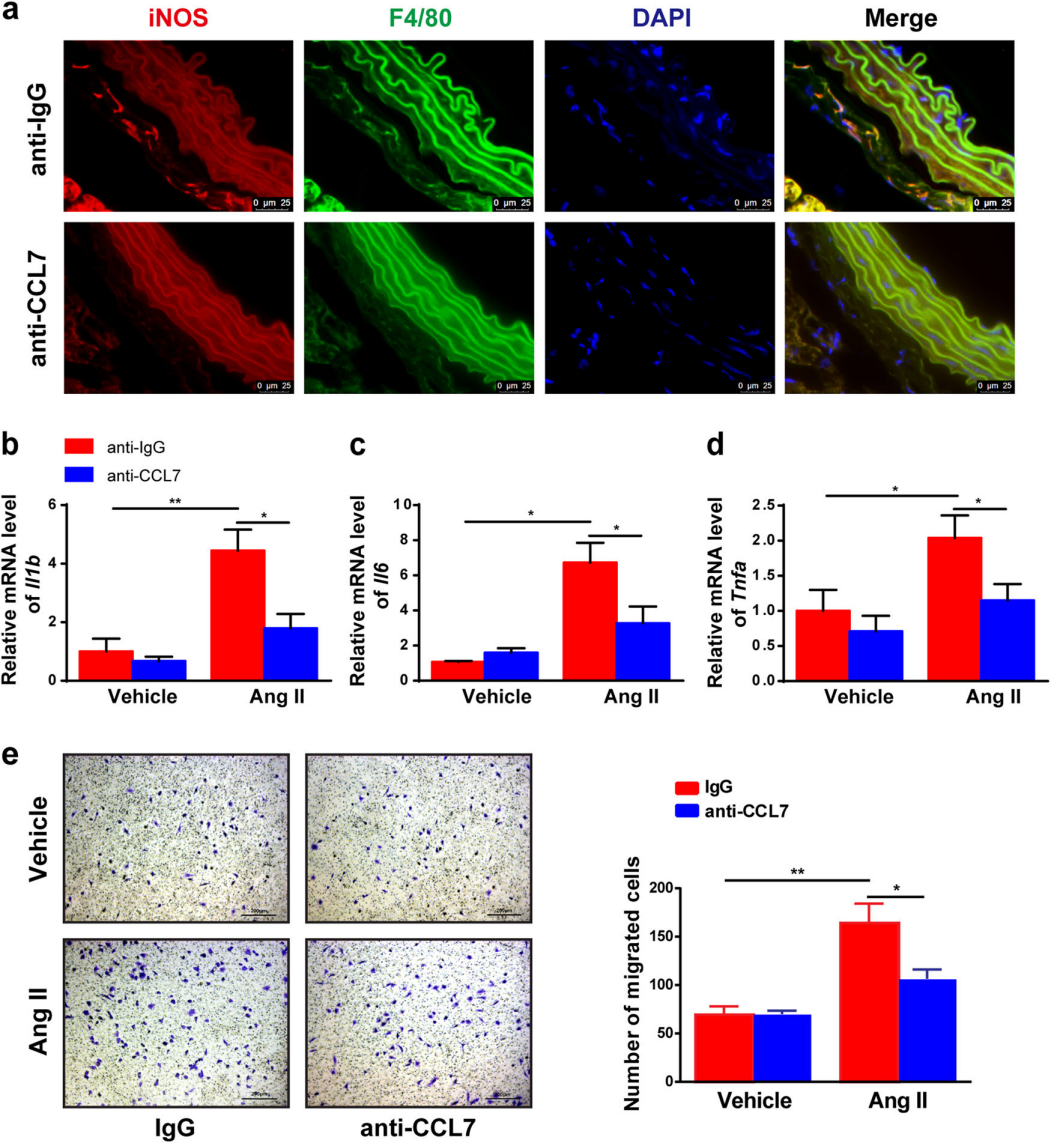
CCL7 neutralization alleviates Ang II-induced M1 macrophage infiltration. Wild-type mice were infused with 1000 ng/kg/min Ang II for 28 days in the present of control IgG (anti-IgG) or CCL7-neutralizing antibody (anti-CCL7). **a** Immunofluorescence analysis of representative cross-sections of mice aortas for iNOS (red) and F4/80 (green) with DAPI counterstaining (blue). The mRNA expression of *Il1b* (**b**), *Il6* (**c**), and *Tnfa* (**d**) in aortas were analyzed by qPCR. *P < 0.05, **P < 0.01, *n* = 6 per group. **e** Chemotaxis assay for BMDM induced by supernatant from vehicle or Ang II-treated wild-type VSMCs in the presence of 8 μg/mL of control IgG or CCL7-neutralizing antibody. *P < 0.05 and **P < 0.01, *n* = 6 (independent experiments)

To clarify the effect of CCL7 neutralization on M1 macrophage infiltration, immunofluorescence analysis of the M1 macrophage markers iNOS and F4/80 were performed in aortic sections. Neutralizing CCL7 decreased Ang II-induced M1 macrophage infiltration (Fig. 7a). Furthermore, *Il1b, Il6*, and *Tnfa* mRNAs were also lower in aortas treated with CCL7-neutralizing antibody (Fig. 7b). In vitro, blockade of CCL7 by neutralizing antibody abolished macrophage migration induced by Ang II-treated VSMCs (Fig. 7c). These data demonstrated that CCL7 neutralization suppresses Ang II-induced M1 macrophage infiltration, thus suggesting that CCL7 is a critical chemokine in mediating macrophage recruitment and vascular remodeling upon Ang II treatment, thus might serve as a potential therapeutic target for vascular remodeling disease.

## Discussion

This study demonstrated that inhibition of HIF1α signaling in VSMCs by genetic disruption in mice attenuated Ang II-induced hypertension and vascular remodeling. *Hif1a* disruption in VSMC reduced Ang II-induced M1 macrophage infiltration into the aortas, and decreased proinflammatory cytokine expression and fibrosis. Mechanistically, CCL7, a chemokine critical for macrophage recruitment, was identified as a novel HIF1α but not HIF2α direct target gene. A CCL7-neutralizing antibody inhibited macrophage chemotaxis induced by Ang II-stimulated VSMCs in vitro. It is important to note that neutralizing CCL7 significantly alleviated Ang II-induced hypertension and vascular remodeling in vivo. Thus, this study provides compelling evidence supporting a strong causative role for VSMC HIF1α and M1 macrophage infiltration in the pathogenesis of arterial hypertension and vascular remodeling. Inhibition of HIF1α or its downstream CCL7 may therefore be a potential therapeutic strategy for treating vascular remodeling-related diseases.

Vascular remodeling is the key pathological basis for many cardiovascular diseases, such as hypertension, atherosclerosis, aortic aneurysm, and restenosis. Numerous studies have demonstrated that Ang II-induced vascular inflammation is a critical step for vascular remodeling. In this study, Ang II treatment significantly increased the expression of proinflammatory cytokine *Il1b*, *Il6*, and *Tnfa* mRNA expression, but this effect was dramatically attenuated by disruption of HIF1α in VSMCs, indicating that VSMC HIF1α plays a key role in Ang II-induced vascular inflammation. This is consistent with a previous report that Ang II-induced medial thickening with VSMC hypertrophy and vascular fibrosis in the aortae was suppressed in HIF1α-deficient mice^20^. In contrast to the present results and earlier studies^20^, another report found that lack of HIF1α in VSMCs increased hypertension in vivo and hyperresponsiveness of resistance vessels to Ang II stimulation ex vivo^21^. This was attributed to the potential effects of HIF1α on peroxisome proliferator-activated receptor γ (PPARγ) that was decreased in VSMC lacking HIF1α. PPARγ activation was found to reduce ATR1 expression in VSMC^21,28^. These data revealed that VSMC HIF1α deficiency increased hypertension and vascular remodeling through the PPARγ-mediated decrease in ATR1. However, the mechanisms by which HIF1α induces PPARγ, and how PPARγ activation decreases ATR1 in cultured VSMC are not known, nor whether this PPARγ pathway occurs in vivo.

It is well established that inflammatory cell (such as T cells and macrophages) infiltration into the vessel wall and their interactions with local cells largely contribute to vascular inflammation and further remodeling process^29^. Interestingly, the present study found that HIF1α deficiency in VSMCs specifically inhibited Ang II-induced M1 macrophage recruitment without affecting T cells or neutrophils, thus highlighting an important role for HIF1α in mediating the interaction between VSMCs and M1 macrophages. The mobilization of monocytes/macrophages from bone marrow to inflammatory sites relies on the chemokine–chemokine receptor interaction^30^. Mechanistically, HIF1α deficiency in VSMCs markedly suppressed the expression of chemokine CCL7. CCL7, also called MCP-3, is an agonist for CCR2 in vivo. It was demonstrated that CCL7 is critical for monocyte mobilization from bone marrow and recruitment to inflammatory sites, since the number of inflammatory monocytes was profoundly reduced in *Ccl7*^−/−^ mice^31^. Several studies suggest that CCL7 may also be involved in vascular pathologies^32,33^. CCL7 can be regulated by many factors, such as IL1β, interferon-α, and interferon-β^34^. In this study, 2% oxygen or the hypoxia mimick CoCl_2_, and Ang II significantly upregulated the *Ccl7* mRNA and CCL7 protein expression in VSMCs. Bioinformatics analysis and ChIP assays identified three functional HREs on the *Ccl7* promoter. Interestingly, *Ccl7* is an HIF1α but not HIF2α target gene, indicating distinct target gene profiles for HIF1α and HIF2α in different cells although they share some common target genes^35^. Neutralizing CCL7 significantly blocked the macrophage chemotaxis by Ang II-treated VSMCs. Moreover, CCL7 blockade attenuated Ang II-induced hypertension and vascular remodeling in vivo, indicating that HIF1α or CCL7 blockade may be a potential means for treating vascular remodeling disease.

Of note, the potential limitation of this study is the application of SM22α-Cre as a tool for studying VSMC functions, as several reports indicated that SM22α-Cre is also expressed in the embryonic heart and some non-muscular cells^36^. However, SM22α-Cre-driven *Hif1a* downregulation was only detected in VSMCs and aortas, but not in heart, spleen, kidney, and adipose tissues, thus supporting the view that VSMCs are the major contributors to the phenotype in SM22α-Cre-driven *Hif1a*-deficient mice. Future rigorous fate-mapping approaches could further expand our knowledge of the physiological consequences of SMC plasticity.

In conclusion, direct evidence is provided that deficiency of HIF1α in VSMCs inhibits Ang II-induced vascular remodeling and blockade of chemokine CCL7, an HIF1α direct target in VSMCs, ameliorates Ang II-induced M1 macrophage recruitment and consequent vascular remodeling. These findings highlight CCL7 as a critical molecule mediating the crosstalk between VSMCs and M1 macrophages during vascular remodeling. This study suggests that HIF1α and CCL7 may serve as a novel therapeutic target to inhibit vascular remodeling.

## Materials and methods

The data that support the findings of this study are available from the corresponding author upon request. Please see the online-only Data Supplement for additional information.

### Animals and treatments

C57BL/6J wild-type mice were purchased from Charlies River Company (Beijing, China). C57BL/6J-background *Hif1a*^fl/fl^^37^ and *Hif2a*^fl/fl^ mice^14,38^ were crossed with SM22a-Cre transgenic mice harboring the Cre recombinase under the control of the murine smooth muscle protein 22α promoter, *SM22α*-Cre (generated by Joachim Herz, University of Texas Southwest Medical Center, and obtained from the Jackson Laboratory)^39^ to generate VSMC-specific *Hif1a*-deficient (*Hif1a*^ΔSMC^) or *Hif2a*-deficient (*Hif2a*^ΔSMC^) mice and their littermate controls. Mice were housed in temperature- and light-controlled rooms with free access to water and pelleted chow ad libium. To establish Ang II-induced vascular remodeling model, 10 week-old male *Hif1a*^ΔSMC^ and the littermate control *Hif1a*^fl/fl^ mice were infused with saline or Ang II at a dosage of 1000 ng/kg/min (Sigma-Aldrich, St. Louis, MO, USA) for 28 days by subcutaneously implanted micro-osmotic pumps (Alzet MODEL 1004; DURECT, Cupertino, CA, USA) as previously described^25^. To detect the hypoxic niche in vivo, mice were injected with 60 mg/kg hypoxyprobe (Hypoxyprobe, MA, USA), a pimonidazole that forms covalent protein adducts in viable hypoxic cells^40,41^, 2h before killing. For neutralizing CCL7 experiment in vivo, the normal IgG (AB-108-C; R&D systems, Minneapolis, MN, USA) or CCL7-neutralizing antibody (AF-456-NA, R&D systems) was administered intravenously at a dose of 2 μg/mice every other day from 1 day before Ang II infusion. Detailed methods are described in the Data Supplement. All animal studies were carried out in accordance with guidelines and approved by Capital Medical University Animal Care and Use Committee.

### Blood pressure measurements

SBP and DBP were measured by a non-invasive tail-cuff sphygmomanometer (BP-98A, Softron, Tokyo, Japan) as described previously^25^.

### Vascular ultrasonic studies

Vascular function was analyzed by echocardiography using a Vevo 2100 console (FUJIFILM VisualSonic, Bothell, WA, USA). Mice were anesthetized with inhaled isoflurane (1% in O_2_). All measurements were obtained from three to six consecutive cardiac cycles, and the averaged values used for analysis. Detailed methods are described in the Data Supplement.

### Vascular relaxation studies

The thoracic aortas were cut into 4 mm segments and gently mounted on force transducers (Power Laboratory; AD Instruments, Bella Vista, Australia) in organ chambers. After stimulation by noradrenaline, vascular responses to increasing concentrations of acetylcholine and sodium nitroprusside (SNP) were recorded. Detailed methods are described in the Data Supplement.

### Histology

Aortas were fixed in 4% paraformaldehyde, embedded in OCT, sectioned into 7 µm, and stained with H&E as described previously^42^. Medium thickness was counted by measuring (perimeter of external medial−perimeter of lumen)/2*π* and calculate the average of each group. Elastin staining was performed with the Gomori’s aldehyde-fuchsin staining method using a commercial kit (Maixin Bio, Fuzhou, China)^43^. Collagen deposition was assessed by Masson’s trichrome staining (Sigma-Aldrich, HT15-1KT) according to the manufacturer’s instruction. All the histology was evaluated by a blinded observer and quantified by Image J software (ImageJ, NIH, Bethesda, MD, USA).

### Flow cytometry

Inflammatory cells in aortas were analyzed by flow cytometry as previously described^25^. Single-cell suspensions were treated with Fc block, washed, and stained with CD45 percpCy5.5 (557235, BD), CD11b FITC (557396, BD), F4/80 BV421 (565411, BD), Ly6G APC (560599, BD), CD206 PE (141706, BD), CD3 BV421 (562600, BD), CD4 V500 (560782, BD), CD8 FITC (553030, BD), CD49b APC (558295, BD), NK1.1 PE (557391, BD), and their homologous isotype-matched negative controls (BD, Franklin Lakes, NJ). In the basis of a live gate, events were acquired on a Fortessa flow cytometer (BD) and analyzed. Detailed methods are described in the Data Supplement.

### Mouse primary VSMCs isolation and treatment

Primary VSMCs were isolated from aortas of 8- to 10-week-old *Hif1a*^fl/fl^ and *Hif1a*^∆SMC^ mice as previously described^43,44^. The purity of VSMCs was over 95% as assessed by α-SMA (Sigma-Aldrich) immunofluorescent staining (Supplementary Fig. 1). Cells from passages 3 to 7 were used for the in vitro studies. VSMCs were treated with vehicle or Ang II (1 μM) for 24 h. The supernatants were collected for CCL7 measurement by enzyme-linked immunosorbent assay (ELISA) or served as chemoattractant for macrophage chemotaxis assays, and the cells were collected for mRNA or protein analysis. To detect whether *Ccl7* mRNA expression was controlled by HIF1α activation, VSMCs were exposed to normoxia or hypoxia (2% O_2_), vehicle, or CoCl_2_ (150 μM) for 6, 12, and 24 h. For rescue study, VSMCs from *Hif1a*^∆SMC^ mice were infected with recombinant lentivirus expressing oxygen-stable HIF1α for 24 h^45^. Detailed methods are described in the Data Supplement.

### Bone marrow-derived macrophage isolation and culture

Bone marrow-derived cells were isolated from the femurs and tibias of adult wild-type mice as described previously^46^. Cells were plated in Dulbecco’s modified Eagle's medium (DMEM) complete medium (10% fetal bovine serum (FBS) and 1% penicillin and streptomycin) and stimulated with murine macrophage colony-stimulating factor (50 ng/mL) for 4 days to allow the differentiation into macrophages.

### Chemotaxis assay

To detect the chemotactic ability of Ang II-treated *Hif1a*^fl/fl^ or *Hif1a*^ΔSMC^ VSMCs on macrophages, chemotaxis assay was performed in 24-well Boyden chambers with 5 µm pore size polycarbonate membranes (Corning, NY, USA). The supernatants from 1 μM Ang II-treated *Hif1a*^fl/fl^ or *Hif1a*^ΔSMC^ VSMCs were added into the bottom chamber as chemoattractant, and 1 × 10^5^ bone marrow-derived macrophages were seeded onto the upper chamber. For the CCL7-neutralizing experiment, 8 μg/mL normal goat IgG control or CCL7-neutralizing antibody was added into the bottom chamber together with the VSMC supernatants. For all chemotaxis assays, after incubation for 6 h at 37 °C, the residual cells on the upper side of the membrane were removed by a cotton swab. The cells that migrated to the lower side of the membrane were fixed with 4% paraformaldehyde, stained with 0.1% crystal violet solution for 5 min, and the cell numbers were counted. Each well represented an independent experiment but not experimental replicate, because the VSMCs from each mouse were cultured separately but not pooled.

### Macrophage-VSMC adhesion assay

To detect the adhesion ability of Ang II-treated *Hif1a*^fl/fl^ or *Hif1a*^ΔSMC^ VSMCs on macrophages, macrophages were resuspended and incubated with 5 μM Calcein-AM (Thermo Fisher Scientific, Waltham, MA USA) for 30 min at 37 °C. Then 5 × 10^4^ Calcein-AM-labeled macrophages were added into 24-well plates with pre-cultured VSMCs in the presence of 1 μM Ang II. After incubation for 1 h at 37 °C, the non-adherent macrophages were washed out by phosphate-buffered saline (PBS) and the residual macrophages were counted under a fluorescent microscope (Nikon Eclipse Ti-U, Tokyo, Japan).

### Enzyme-linked immunosorbent assay analysis

The concentrations of CCL7 in the supernatant from Ang II-treated VSMCs were measured by mouse MCP3 ELISA Kit (CCL7) (ab205571, Abcam) according to the manufacturer’s instructions.

### RNA analysis

Total RNA was extracted from aortic tissues or cultured VSMCs using Trizol reagent (Invitrogen, Carlsbad, CA, USA). The first strand of cDNA was synthesized from 2 µg of RNA using the GoScript^TM^ Reverse Transcription System (Promega, Mannheim, Germany). Real-time quantitative PCR was performed with SYBR Green I (Takara, Shiga, Japan) using primers listed in Supplementary Table 1 on an iCyclerIQsystem (Bio-Rad, Hercules, CA, USA). The relative gene expression levels were analyzed with the 2^−ΔΔCt^ method. *Actb* mRNA was used as a control.

### Microarray

VSMCs from *Hif1a*^fl/fl^ and *Hif1a*^ΔSMC^ mice were treated with 150 μM CoCl_2_ for 24 h. The total RNA was extracted in Trizol reagent (Invitrogen, Carlsbad, CA, USA) and detected by CapitalBio Technology (Beijing, China). Detailed methods are described in the Data Supplement.

### Western blotting

Whole-cell lysate was extracted from VSMCs using RIPA Lysis Buffer (Applygen, China). Protein concentration of the samples was measured by a microplate protein assay, and equal amounts of protein per sample and known molecular weight markers were subjected to SDS-polyacrylamide gel electrophoresis (SDS-PAGE). Membranes were incubated with primary antibodies against HIF1α (1:1000, NB100-105; Novus Biologicals) and ACTB (1:5000, 66009-1; Proteintech, IL 60018, USA) overnight at 4 °C, then with anti-mouse secondary antibody (1:4000, 7076S; Cell Signaling, MA, USA) conjugated with horseradish peroxidase for 1 h at room temperature. Immunodetection analyses were performed using a Chemiluminescence HRP Substrate Kit (Millipore, Massachusetts, USA). Detailed methods are described in the Data Supplement.

### Luciferase assays

The Ccl7 promoter was cloned using primers listed below (Forward: 5′-ATACATGAGCTCCCTATTTCCACCTTTGTCTGCTA-3′; Reverse: 5′-ATACATCTCGAGCCCAAAGCATTCTTTCCAAGTC-3′). The *Ccl7* promoter was then digested and cloned into the pGL3-basic vector (Promega). The sequences were confirmed via sanger sequencing by the DNA Sequencing Core Facility at the University of Michigan. HEK293T human embryonic kidney cells were cultured at 37 °C in 5% carbon dioxide and 21% oxygen. DMEM was used to culture the cells and was supplemented with 10% FBS and 1% antibiotic/antimycotic (1 unit/mL of penicillin, 1 mg/mL of streptomycin, and 2.5 ng/mL of amphotericin B; Life Technologies). Celeste Simon at the University of Pennsylvania School of Medicine provided the oxygen-stable HIF1TM. The HIF1α constructs contained mutations at the critical proline residues, rescuing them from proteasomal degradation resulting in their stabilization. *Ccl7* promoter luciferase reporters were then co-transfected with HIF1α or an empty vector control with polyethylenimine (PEI; Polyscience). Cells were incubated for 48 h before lysis in luciferase lysis buffer (25 mM Tris-phosphate (pH 7.8), 2 mM DTT, 2 mM 1,2-diaminocyclohexane-*N*,*N*,*N*′,*N*’-tetraacetic acid, 10% glycerol, 1% Triton® X-100). Fifteen microliters of lysate were added to a 96-well opaque white plate and 25 μL of Luciferase assay buffer (4.8 mL tris 0.11 mM pH 7.8, 50 μL sodium Luciferin 100 mM, 60 μL ATP 200 mM, and 60 μL MgCl_2_ 1 M) and luminescence was measured. Fifty microliters of lysate was added to a clear 96-well plate with 50 μL of 2-nitrophenyl β-d-galactopyranoside (ONPG; Sigma-Aldrich) for the β-galactosidase assay and absorbance was measured at 420 nm. Luciferase values were normalized to β-galactosidase values.

### ChIP assays

*Hif1a*^fl/fl^ or *Hif1a*^ΔSMC^ VSMCs treated with Ang II (1 μM) for 24 h were crosslinked in 1% formaldehyde in 1× PBS for 10 min. ChIP assays were performed for HIF1α (2 μg/IP, NB100-105; Novus Biologicals) or HIF2α (2 μg/IP, NB100-122; Novus Biologicals) using Simple ChIP Plus Kit (Cell Signaling Technology, Danvers, MA, USA) as previously described^14^. The primers for ChIP assays are listed in Supplementary Table 1.

### Statistical analysis

The mean values ± S.E.M. were calculated and plotted using GraphPad Prism 7 software (GraphPad Software, San Diego, California, USA). Comparisons between two groups were performed using two-tailed unpaired Student’s *t*-test. Differences between multiple groups with one variable were determined using one-way analysis of variance (one-way ANOVA) followed by Bonferroni’s post-hoc test. To compare multiple groups with more than one variable, two-way ANOVA followed by Bonferroni’s post-hoc test was used. *P* < 0.05 was considered statistically significant.

## Supplementary information

Supplemental Figure 1

Supplemental Figure 2

Supplemental Figure 3

Supplemental Figure 4

Supplemental Table 1

Supplemental Methods

## References

[1] Collaborators, G. B. D. R. F. (2016). Global, regional, and national comparative risk assessment of 79 behavioural, environmental and occupational, and metabolic risks or clusters of risks, 1990-2015: a systematic analysis for the Global Burden of Disease Study 2015. Lancet.

[2] McMaster WG, Kirabo A, Madhur MS, Harrison DG (2015). Inflammation, immunity, and hypertensive end-organ damage. Circ Res.

[3] Viel EC, Lemarie CA, Benkirane K, Paradis P, Schiffrin EL (2010). Immune regulation and vascular inflammation in genetic hypertension. Am J Physiol Heart Circ Physiol.

[4] Capers Qt (1997). Monocyte chemoattractant protein-1 expression in aortic tissues of hypertensive rats. Hypertension.

[5] Rudemiller NP, Crowley SD (2017). The role of chemokines in hypertension and consequent target organ damage. Pharmacol Res.

[6] Mateo T (2006). Angiotensin II-induced mononuclear leukocyte interactions with arteriolar and venular endothelium are mediated by the release of different CC chemokines. J Immunol.

[7] Semenza GL (2012). Hypoxia-inducible factors in physiology and medicine. Cell.

[8] Semenza GL (1996). Hypoxia response elements in the aldolase A, enolase 1, and lactate dehydrogenase A gene promoters contain essential binding sites for hypoxia-inducible factor 1. J Biol Chem.

[9] Semenza GL (2014). Oxygen sensing, hypoxia-inducible factors, and disease pathophysiology. Ann Rev Pathol.

[10] Semenza GL (2009). Involvement of oxygen-sensing pathways in physiologic and pathologic erythropoiesis. Blood.

[11] Jaakkola P (2001). Targeting of HIF-alpha to the von Hippel-Lindau ubiquitylation complex by O_2_-regulated prolyl hydroxylation. Science.

[12] Xia X (2009). Integrative analysis of HIF binding and transactivation reveals its role in maintaining histone methylation homeostasis. Proc Nat Acad Sci USA.

[13] Mole DR (2009). Genome-wide association of hypoxia-inducible factor (HIF)-1alpha and HIF-2α DNA binding with expression profiling of hypoxia-inducible transcripts. J Biol Chem.

[14] Qu A (2011). Hypoxia-inducible transcription factor 2α promotes steatohepatitis through augmenting lipid accumulation, inflammation, and fibrosis. Hepatology.

[15] Hanze J, Weissmann N, Grimminger F, Seeger W, Rose F (2007). Cellular and molecular mechanisms of hypoxia-inducible factor driven vascular remodeling. Thromb Haemost.

[16] Jain T, Nikolopoulou EA, Xu Q, Qu A (2018). Hypoxia inducible factor as a therapeutic target for atherosclerosis. Pharmacol Ther.

[17] Nakayama T (2013). Role of macrophage-derived hypoxia-inducible factor (HIF)-1alpha as a mediator of vascular remodelling. Cardiovasc Res.

[18] Kurobe H (2010). Role of hypoxia-inducible factor 1α in T cells as a negative regulator in development of vascular remodeling. ATVB.

[19] Lambert CM, Roy M, Robitaille GA, Richard DE, Bonnet S (2010). HIF-1 inhibition decreases systemic vascular remodelling diseases by promoting apoptosis through a hexokinase 2-dependent mechanism. Cardiovasc Res.

[20] Imanishi M (2014). Smooth muscle cell-specific Hif-1α deficiency suppresses angiotensin II-induced vascular remodelling in mice. Cardiovasc Res.

[21] Huang Y (2013). Hypoxia-inducible factor-1α in vascular smooth muscle regulates blood pressure homeostasis through a peroxisome proliferator-activated receptor-γ-angiotensin II receptor type 1 axis. Hypertension.

[22] Sui X, Wei H, Wang D (2015). Novel mechanism of cardiac protection by valsartan: synergetic roles of TGF-β1 and HIF-1α in Ang II-mediated fibrosis after myocardial infarction. J Cell Mol Med.

[23] Yang L (2016). Silencing of hypoxia inducible factor-1α gene attenuated angiotensin-induced abdominal aortic aneurysm in apolipoprotein E-deficient mice. Atherosclerosis.

[24] Medley TL, Kingwell BA, Gatzka CD, Pillay P, Cole TJ (2003). Matrix metalloproteinase-3 genotype contributes to age-related aortic stiffening through modulation of gene and protein expression. Circ Res.

[25] Wang L (2016). Genetic and pharmacologic inhibition of the chemokine receptor CXCR2 prevents experimental hypertension and vascular Dysfunction. Circulation.

[26] Stenmark KR, Tuder RM, El Kasmi KC (2015). Metabolic reprogramming and inflammation act in concert to control vascular remodeling in hypoxic pulmonary hypertension. J Appl Physiol.

[27] Martynowicz H, Janus A, Nowacki D, Mazur G (2014). The role of chemokines in hypertension. Adv Clin Exp Med.

[28] Jefferson AL (2018). Higher aortic stiffness is related to lower cerebral blood flow and preserved cerebrovascular reactivity in older adults. Circulation.

[29] Tellides G, Pober JS (2015). Inflammatory and immune responses in the arterial media. Circ Res.

[30] Kratofil RM, Kubes P, Deniset JF (2017). Monocyte conversion during inflammation and injury. ATVB.

[31] Kuo CH, Collins AM, Boettner DR, Yang Y, Ono SJ (2017). Role of CCL7 in type I hypersensitivity reactions in murine experimental allergic conjunctivitis. J Immunol.

[32] Schenk S (2007). Monocyte chemotactic protein-3 is a myocardial mesenchymal stem cell homing factor. Stem Cells.

[33] Maddaluno M (2011). Monocyte chemotactic protein-3 induces human coronary smooth muscle cell proliferation. Atherosclerosis.

[34] Jacobsson B, Holst RM, Andersson B, Hagberg H (2005). Monocyte chemotactic protein-2 and -3 in amniotic fluid: relationship to microbial invasion of the amniotic cavity, intra-amniotic inflammation and preterm delivery. Acta Obstet Gynecol Scand.

[35] Semenza GL (2014). Hypoxia-inducible factor 1 and cardiovascular disease. Annu Rev Physiol.

[36] Chakraborty R (2019). Promoters to study vascular smooth muscle. ATVB.

[37] Tomita S (2003). Defective brain development in mice lacking the Hif-1α gene in neural cells. Mol Cell Biol.

[38] Gruber M (2007). Acute postnatal ablation of Hif-2alpha results in anemia. Proc Natl Acad Sci USA.

[39] Holtwick R (2002). Smooth muscle-selective deletion of guanylyl cyclase-A prevents the acute but not chronic effects of ANP on blood pressure. Proc Natl Acad Sci USA.

[40] Aguilera, K. Y. & Brekken, R. A. Hypoxia Studies with Pimonidazole in vivo. *Bio Protoc* **4**, e1254 (2014). 10.21769/bioprotoc.1254 PMC4956402 27453908

[41] Hofer SO (2005). The use of pimonidazole to characterise hypoxia in the internal environment of an in vivo tissue engineering chamber. Br J Plast Surg.

[42] Wang L (2014). Inhibition of Toll-like receptor 2 reduces cardiac fibrosis by attenuating macrophage-mediated inflammation. Cardiovasc. Res..

[43] Jia LX (2015). Mechanical stretch-induced endoplasmic reticulum stress, apoptosis and inflammation contribute to thoracic aortic aneurysm and dissection. J Pathol.

[44] Golovina VA, Blaustein MP (2006). Preparation of primary cultured mesenteric artery smooth muscle cells for fluorescent imaging and physiological studies. Nat Protoc.

[45] Dioum EM (2009). Regulation of hypoxia-inducible factor 2alpha signaling by the stress-responsive deacetylase sirtuin 1. Science.

[46] Wang C (2017). Macrophage-derived mir-155-containing exosomes suppress fibroblast proliferation and promote fibroblast inflammation during cardiac injury. Mol Ther.

