# Supplementary material for: Hypoxia inducible factor 1α in vascular smooth muscle cells promotes angiotensin II-induced vascular remodeling via activation of CCL7-mediated macrophage recruitment
Source: Cell Death Dis. 2019 Jul 18;10(8):544. doi: 10.1038/s41419-019-1757-0 (PMC6639417; doi:10.1038/s41419-019-1757-0)
Supplement: Supplementary file 1 — Supplemental Figure 1 [file 41419_2019_1757_MOESM1_ESM.pdf]

## Supplementary figures

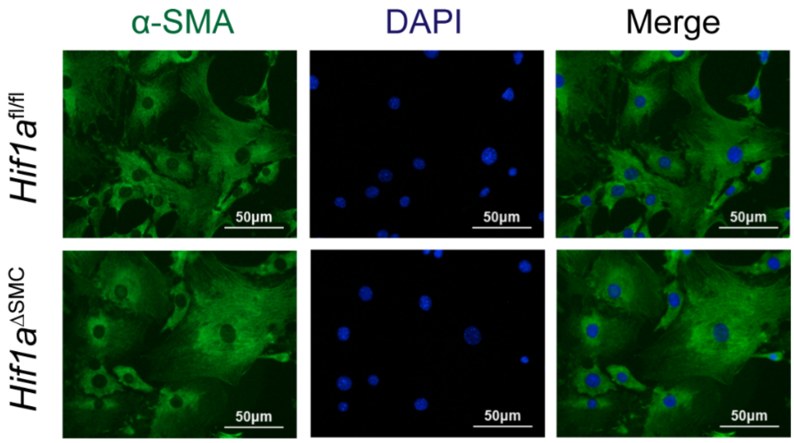


**Supplementary Fig. 1. Purity identification of VSMCs.** Immunofluorescence staining was used to detect the expression of α-SMA, a specific marker of smooth muscle cells, in primary cultured VSMCs of *Hif1a*$^{fl/fl}$ and *Hif1a*$^{\Delta SMC}$ mice.
