# Supplementary material for: Hypoxia inducible factor 1α in vascular smooth muscle cells promotes angiotensin II-induced vascular remodeling via activation of CCL7-mediated macrophage recruitment
Source: Cell Death Dis. 2019 Jul 18;10(8):544. doi: 10.1038/s41419-019-1757-0 (PMC6639417; doi:10.1038/s41419-019-1757-0)
Supplement: Supplementary file 2 — Supplemental Figure 2 [file 41419_2019_1757_MOESM2_ESM.pdf]

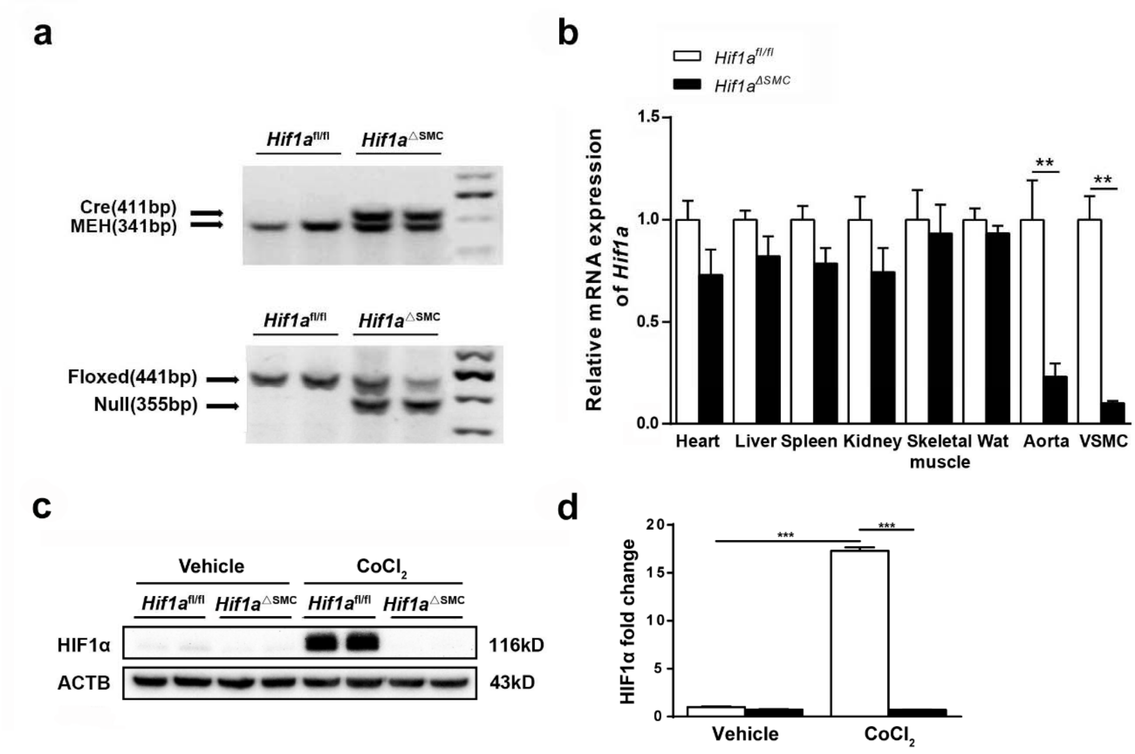

Supplementary Fig. 2. Identification of VSMC-specific HIF1α-deficient mice. a Polymerase chain reaction (PCR) analysis showed the *Hif1a* and *Cre* genes and loxP sites in DNA extracted from *Hif1a*$^{fl/fl}$ and *Hif1a*$^{\Delta SMC}$ mouse tails. **b** *Hif1a* mRNA expression in mouse tissues and VSMCs. ***P*<0.01, n=6 per group. **c** western blotting analysis of HIF1α protein in VSMCs treated with 150 μM $CoCl_2$ for 6 hours and quantification **d**. ****P*<0.001, n=3 ((independent experiments), statistical significance was determined by the unpaired t test.
