# Supplementary material for: Hypoxia inducible factor 1α in vascular smooth muscle cells promotes angiotensin II-induced vascular remodeling via activation of CCL7-mediated macrophage recruitment
Source: Cell Death Dis. 2019 Jul 18;10(8):544. doi: 10.1038/s41419-019-1757-0 (PMC6639417; doi:10.1038/s41419-019-1757-0)
Supplement: Supplementary file 3 — Supplemental Figure 3 [file 41419_2019_1757_MOESM3_ESM.pdf]

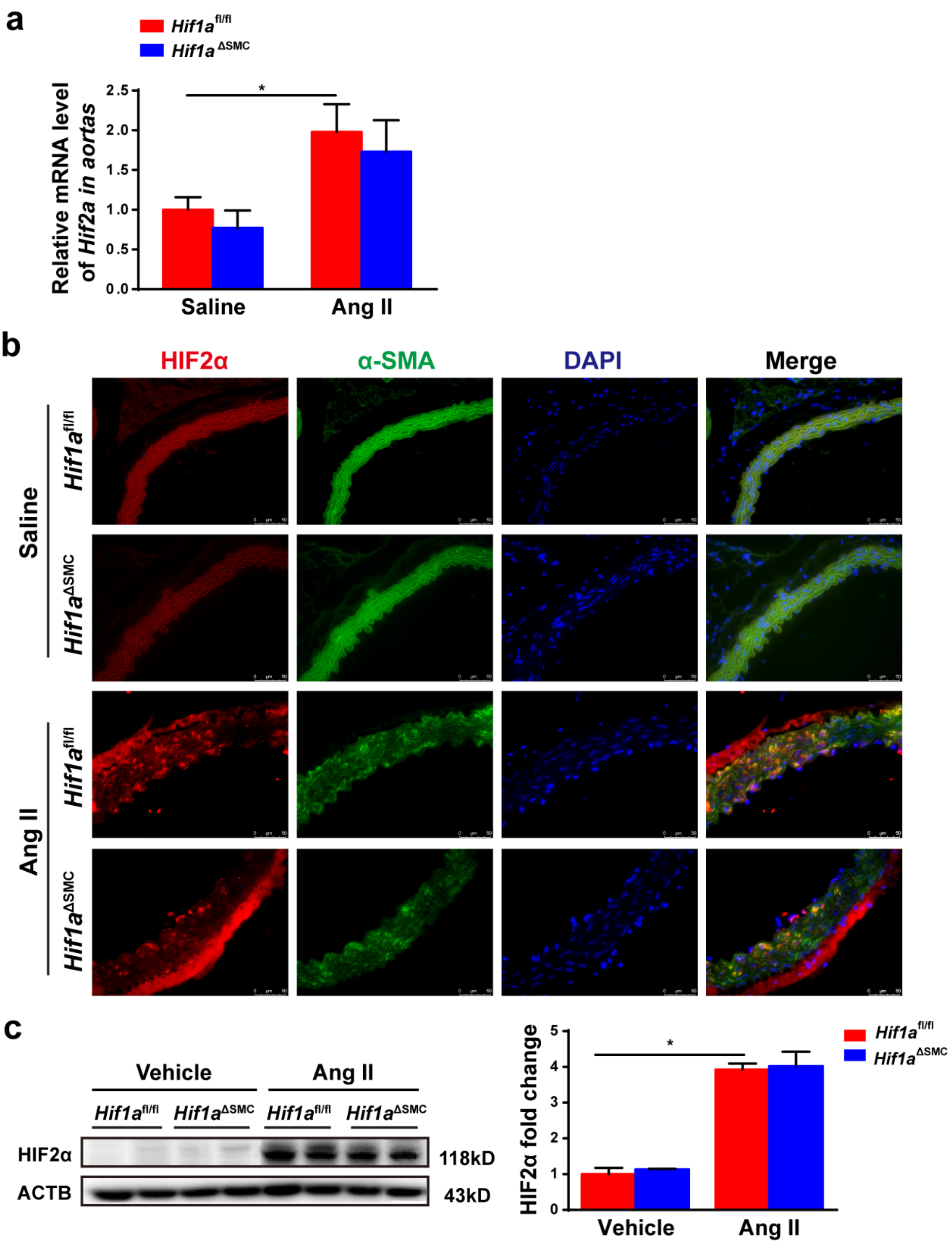

Supplementary Fig. 3. Deficiency of HIF1α in VSMCs does not affect Ang II-induced HIF2α expression in aortas. *Hif1a*$^{fl/fl}$ and *Hif1a*$^{\Delta SMC}$ mice were infused with saline or 1000 ng/kg/min Ang II for 28 days. **a** *Hif2a* mRNA level in aortas were measured by qPCR. **P*<0.05, n=6 per group. **b** Immunofluorescent staining for HIF2α (in red) and α-SMA (in green) with DAPI counterstaining (in blue) in aortic sections from saline or Ang II-infused *Hif1a*$^{fl/fl}$ and *Hif1a*$^{\Delta SMC}$ mice. **c** *Hif1a*$^{fl/fl}$ and *Hif1a*$^{\Delta SMC}$ VSMCs were treated with vehicle or Ang II (1 μM) for 24 hours and HIF2α protein level was detected by Western blot. *p<0.05, n=3 per group.
