# Supplementary material for: Hypoxia inducible factor 1α in vascular smooth muscle cells promotes angiotensin II-induced vascular remodeling via activation of CCL7-mediated macrophage recruitment
Source: Cell Death Dis. 2019 Jul 18;10(8):544. doi: 10.1038/s41419-019-1757-0 (PMC6639417; doi:10.1038/s41419-019-1757-0)
Supplement: Supplementary file 4 — Supplemental Figure 4 [file 41419_2019_1757_MOESM4_ESM.pdf]

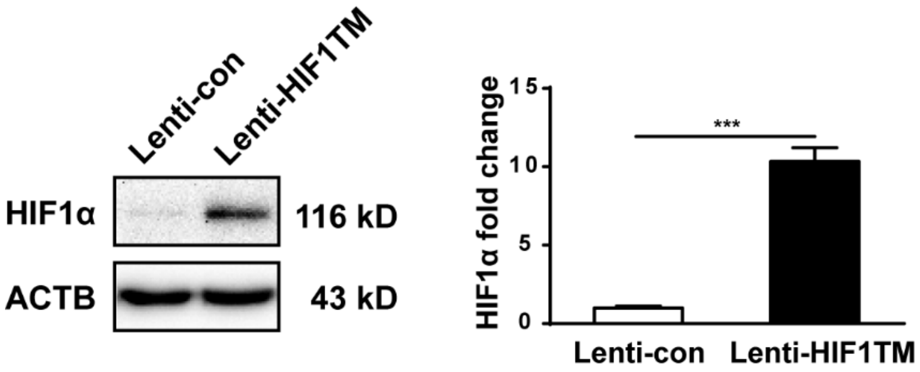

Supplementary Fig. 4. Overexpression of HIF1α in VSMCs by lenti-virus. Western blotting analysis of HIF1α protein in VSMCs infected with lentivirus expressing oxygen-stable HIF1α (Lenti-HIF1TM) for 24 hours and quantification.
