# Supplementary material for: Hypoxia inducible factor 1α in vascular smooth muscle cells promotes angiotensin II-induced vascular remodeling via activation of CCL7-mediated macrophage recruitment
Source: Cell Death Dis. 2019 Jul 18;10(8):544. doi: 10.1038/s41419-019-1757-0 (PMC6639417; doi:10.1038/s41419-019-1757-0)
Supplement: Supplementary file 5 — Supplemental Table 1 [file 41419_2019_1757_MOESM5_ESM.pdf]

Supplementary Table 1. Primer list

qPCR	Primer Sequence (5'-3')
Il1b	F: TGTGAAATGCCACCTTTTGA<br>R: GGTCAAAGGTTTGGAAGCAG
Il6	F: ACCAGAGGAAATTTTCAATAGGC<br>R: TGATGCACTTGCAGAAAACA
Tnfa	F: AGGGTCTGGGCCATAGAACT<br>R: CCACCACGCTCTTCTGTCTAC
Mcp1	F: CCTGCTGTTCACAGTTGCC<br>R: ATTGGGATCATCTTGCTGGT
Hif1a	F: ATAGCTTCGCAGAATGCTCAGA<br>R: CAGTCACCTGGTTGCTGCAA
Hif2a	F: TGAGTTGGCTCATGAGTTGCC<br>R: GGAGCTTATGTGTCCGAAGGA
Actb	F: ATGGAGGGGAATACAGCCC<br>R: TTCTTTGCAGCTCCTTCGTT
Cxcl10	F: ATGACGGGCCAGTGAGAATG<br>R: TCGTGGCAATGATCTCAACAC
Cxcl5	F: AGCTGCGTTGTGTTTGCTTA<br>R: CAGTTTAGCTATGACTTCCACCG
Cxcl14	F: GTGGACGGGTCCAAGTGTAA<br>R: CCTCGCAGTGTGGGTACTTT
Ccl7	F: CCCTGGGAAGCTGTTATCTTCAA<br>R: CTCGACCCACTTCTGATGGG
Ccl1	F: ACCATGAAACCCACTGCCAT<br>R: GTAAGCATGCTCTTGCTGTCAA
Ccl24	F: CAAGGCAGGGGTCATCTTCA<br>R: ATGTGCCTCTGAACCCACAG
Ccl26	F: GCTATGTCCTGCTGCCCTAA<br>R: TAAAGAATATCACACCGTCACTGG
Cxcr3	F: CCCAACCACAAGTGCCAAAG<br>R: TCACTAACCTCAAGGTACATGGC
**Genotyping PCR**	

Hif1a$^{fl/fl}$	H1: CTGTCTTCCCTGCTTAGGTCTTTCTAAC<br>H2: GAGATGGAGAAGGAGGTTAGTGTATCC<br>H3: ACGTTGGCTCATGGTGTACTTTG
CRE	A: GCGGTCTGGCAGTAAAAACTATC<br>B: GTGAAACAGCATTGCTGTCACTT<br>C: CTAGGCCACAGAATTGAAAGATCT<br>D: GTAGGTGGAAATTCTAGCATCATCC

ChIP PCR

HRE1	F: CCTATTTCCACCTTTGTCTGCTA<br>R: CATTCCACATTGCCAGTCTTTG
HRE2	F: GGCAGTTGCTCAAACATAGTG<br>R: TCCCAGAAATATTGTCCAGAAGG
HRE3	F: AACATGTCTGCGGGTTACTT<br>R: CCCAAAGCATTCTTTCCAAGTC
