# Supplementary material for: Hypoxia inducible factor 1α in vascular smooth muscle cells promotes angiotensin II-induced vascular remodeling via activation of CCL7-mediated macrophage recruitment
Source: Cell Death Dis. 2019 Jul 18;10(8):544. doi: 10.1038/s41419-019-1757-0 (PMC6639417; doi:10.1038/s41419-019-1757-0)
Supplement: Supplementary file 6 — Supplemental Methods [file 41419_2019_1757_MOESM6_ESM.pdf]

Supplementary material and methods

Animals and Treatments

C57BL/6J-background *Hif1a*$^{fl/fl}$ [1] and *Hif2a*$^{fl/fl}$ mice[2] were crossed with mice harboring the Cre recombinase under the control of the murine smooth muscle protein 22α promoter, *SM22α*-Cre (generated by Joachim Herz, University of Texas Southwest Medical Center, and obtained from the Jackson Laboratory)[3] to generate VSMC-specific *Hif1a*-deficient (*Hif1a*$^{\Delta SMC}$) or *Hif2a*-deficient (*Hif2a*$^{\Delta SMC}$) mice and their littermate controls. Mice were housed in temperature- and light-controlled rooms with free access to water and pelleted *chow ad libium*. All animal studies were carried out in accordance with guidelines and approved by Capital Medical University Animal Care and Use Committee. To establish Ang II-induced vascular remodeling model, 10-week-old male *Hif1a*$^{\Delta SMC}$ and the littermate control *Hif1a*$^{fl/fl}$ mice were infused with saline or Ang II at a dosage of 1000 ng/kg/min (Sigma-Aldrich, St. Louis, MO, USA) for 28 days by subcutaneously implanted micro-osmotic pumps (Alzet MODEL 1004, DURECT, Cupertino, CA, USA) as previously described[4]. To detect the hypoxic niche *in vivo*, PBS or Ang II-infused mice were injected with 60 mg/kg hypoxyprobe (Hypoxyprobe, Burlington, USA), a pimonidazole that forms covalent protein adducts in viable hypoxic cells,[5, 6] two hours before killing. For the CCL7 neutralizing experiment *in vivo*, the normal IgG or CCL7 neutralizing antibody was administered intravenously at a dose of 2 μg/mice every other day from one day before Ang II infusion until 28 days. The detailed information for the IgG control is: normal goat IgG control (AB-108-C, R&D systems, Minneapolis, MN, USA), polyclonal goat IgG (source). The detailed information for CCL7 neutralizing antibody is: mouse CCL7/MCP-3/MARC antibody (AF-456-NA, R&D systems), polyclonal goat IgG (source), mouse (species reactivity).

Vascular Ultrasonic Studies

To assess aortic distensibility and pulse wave velocity (PWV), an electrocardiogram (ECG-gated) image was obtained for the longitudinal aorta and processed using VevoVasc software. Distensibility graph was evaluated as a time plot of D(t)=7.5*[(Area(t)-Area_dia)/Area_dia]/(pressure_sys-pressure_dia). Distensibility parameter is evaluated as d=7.5*[(Area_sys-Area_dia)/Area_dia]/(pressure_sys-pressure_dia). PWV (m/sec) was calculated as distance traveled across the aorta (m) divided by time delay in onset of velocity waves (seconds)[7].

Vascular Relaxation Studies

Intact aortas were gently isolated from mice and stripped of adventitial fat. Four mm segments of the thoracic aorta were mounted on force transducers gently (Power lab, AD Instruments, Germany) in organ chambers filled with Krebs-Henseleit solution (37°C, pH 7.2-7.4, containing 120 mM NaCl, 5.5 mM KCl, 2.5 mM $CaCl_2$, 1.2 mM $MgCl_2 \cdot 6H_2O$, 1.2 mM Na_2HPO_4, 20 mM $NaHCO_3$, 0.03 mM EDTA-Na_2, 10 mM Glucose bubbled with Carbogen gas containing 95% O_2 and 5% CO_2). Vessels were equilibrated for 1 hour before the stimulation of vaso-activity by 60% of the maximal KCl solution (37°C, pH 7.38-7.40, containing 5.248 g NaCl, 4.473 g KCl, 0.244 g $MgCl_2 \cdot 6H_2O$, 0.277 g $CaCl_2$, 2 g glucose and 1.1915 g Hepes in 1000 ml H_2O). To test the relaxation ability of the aorta, the aorta were stimulated to contract with 1*10-6 M Noradrenalin Elisa (NE), and then applied a concentration gradient ($1*10^{-9}$ M, $1*10^{-8}$ M, $1*10^{-7}$ M, $1*10^{-6}$ M, $1*10^{-5}$ M, $1*10^{-4}$ M) acetylcholine (ACh) and sodium nitroprusside (SNP) to relax the aorta.

Immunofluorescence
For immunofluorescence analysis, sections were blocked by 10% normal goat serum and incubated with primary antibodies against α-SMA (1:200, Abcam, Cambridge, UK), HIF1α (1:200, Novus Biologicals, Centennial, CO, USA), HIF2α (1:200, Novus Biologicals), iNOS (1:200, Abcam) and F4/80 (1:200, Abcam) overnight at 4℃, then incubated with FITC-conjugated secondary antibodies (ZF-0312, Zsbio, Beijing, China) at room temperature for 1 hour. All sections were mounted with DAPI (Invitrogen, Carlsbad, CA). All images were obtained by Leica DFC365 FX microscope (Leica, Germany), and further analyzed using ImagePro Plus software (Media Cybernetics, Rockville, MD, USA). All the measurements were performed in a double-blind manner by two different researchers.

Flow Cytometry
Aortic vessels were cleaned of perivascular adipose tissue, minced, and digested with aorta dissociation enzyme solution, which was composed of 125 U/ml collagenase type XI (C7657, Sigma), 60 U/ml hyaluronidase type 1 (H3506, Sigma), 60 U/ml DNase I (D5025-150KU, Sigma), and 450 U/ml collagenase type I (C0130, Sigma). Single-cell suspensions were treated with Fc block, washed, and stained with CD45 percpCy5.5 (557235, BD), CD11b FITC(557396, BD), F4/80 BV421 (565411, BD), Ly6G APC (560599, BD), CD206 PE (141706, BD), CD3 BV421 (562600, BD), CD4 V500 (560782, BD), CD8 FITC (553030, BD), CD49b APC (558295, BD), NK1.1 PE (557391, BD), and their homologous isotype-matched negative controls (BD, Franklin Lakes, NJ). On the basis of a live gate, events were acquired on a Fortessa flow cytometer (BD) and analyzed.[4]

Mouse Primary VSMCs Isolation
Primary VSMCs were isolated from aortas of 8- to 10-week-old *Hif1a*$^{fl/fl}$, *Hif1a*$^{\Delta SMC}$, *Hif2a*$^{fl/fl}$/*ApoE*$^{-/-}$, and *Hif2a*$^{\Delta SMC}$/*ApoE*$^{-/-}$ mice. Briefly, mice were killed by CO_2 inhalation and the aorta dissected and digested with 347 U/mg type II collagenase (Worthington, Lakewood, NJ, USA) at 37°C for 30 min to remove the adventitia under microscopic guidance. Then the aortas were opened longitudinally and the endothelium removed by gently rubbing the intima with a sterile cotton-tipped applicator. The aortas were minced with scissors followed by a further digestion with a mixture of 347 U/mg collagenase II and 6 U/mg elastase IV (Sigma-Aldrich) for 30 min at 37°C. Cells were then cultured with smooth muscle cell medium (SMCM) (ScienCell, CA, USA) supplemented with 10% fetal bovine serum (FBS), 1% smooth muscle growth supplement (SMGS), and 1% penicillin streptomycin (PS). VSMCs from passage 3 to 7 were used for all *in vitro* experiments.

Microarray
Total RNA was extracted from VSMCs by using the Trizol reagent (Invitrogen, Carlsbad, CA, USA). The purity and concentration of RNA were determined from OD260/280 readings using spectrophotometer (NanoDrop ND-1000). RNA integrity was determined by 1% formaldehyde denaturing gel electrophoresis. The cDNAs were hybridized to an Agilent 44 K mouse 60-peptide oligomer microarray (Agilent Technologies, Santa Clara, CA). The data were analyzed for summarization, normalization and quality control by GeneSpring software V13.0 (Agilent). To select the differentially expressed genes, threshold values of ≥2 and ≤−2-fold change and a T-test p value of 0.05 were set. CLUSTER 3.0 software were applied for further

analyses with hierarchical clustering with average linkage. Finally, tree visualization was performed by Java Treeview (Stanford University School of Medicine, Stanford, CA, USA). The data have been uploaded to the GEO repository (with number GSE119226).

Western blotting

Whole-cell lysate was extracted from VSMCs using RIPA Lysis Buffer (Applygen, China) containing protease and phosphatase inhibitors (Thermo, USA). Protein concentration of the samples was measured by a microplate protein assay, and equal amounts of protein per sample and known molecular weight markers were subjected to SDS-polyacrylamide gel electrophoresis (SDS-PAGE). Membranes were incubated with primary antibodies against HIF1α (1:1000, NB100-105, Novus Biologicals) and ACTB (1:5000, 66009-1-Ig, Proteintech, IL 60018, USA) overnight at 4℃, then with anti-mouse secondary antibody (1:4000, 7076S, Cell Signaling, MA, USA) conjugated with horseradish peroxidase for 1 hour at room temperature and washed three times with TBST. Immunodetection analyses were performed using a Chemiluminescence HRP Substrate Kit (Millipore, Massachusetts, USA). ACTB protein was used as a control.

Statistical analysis

The mean values ± S.E.M. were calculated and plotted using GraphPad Prism 7 software (GraphPad Software, San Diego, California, USA). Comparisons between two groups were performed using two-tailed unpaired Student's t-test. Differences between multiple groups with one variable were determined using one-way analysis of variance (One-way ANOVA) followed by Bonferroni's post-hoc test. To compare multiple groups with more than one variable, two-way ANOVA followed by Bonferroni's post-hoc test was used. $P<0.05$ was considered statistically significant.

References

1. Tomita S, Ueno M, Sakamoto M, Kitahama Y, Ueki M, Maekawa N, Sakamoto H, Gassmann M, Kageyama R, Ueda N, Gonzalez FJ and Takahama Y. Defective brain development in mice lacking the Hif-1alpha gene in neural cells. *Mol Cell Biol*. 2003;23:6739-49.
2. Qu A, Taylor M, Xue X, Matsubara T, Metzger D, Chambon P, Gonzalez FJ and Shah YM. Hypoxia-inducible transcription factor 2alpha promotes steatohepatitis through augmenting lipid accumulation, inflammation, and fibrosis. *Hepatology*. 2011;54:472-83.
3. Holtwick R, Gotthardt M, Skryabin B, Steinmetz M, Potthast R, Zetsche B, Hammer RE, Herz J and Kuhn M. Smooth muscle-selective deletion of guanylyl cyclase-A prevents the acute but not chronic effects of ANP on blood pressure. *Proceedings of the National Academy of Sciences of the United States of America*. 2002;99:7142-7.
4. Wang L, Zhao XC, Cui W, Ma YQ, Ren HL, Zhou X, Fassett J, Yang YZ, Chen Y,

Xia YL, Du J and Li HH. Genetic and Pharmacologic Inhibition of the Chemokine Receptor CXCR2 Prevents Experimental Hypertension and Vascular Dysfunction. *Circulation*. 2016;134:1353-1368.
5. Aguilera KY and Brekken RA. Hypoxia Studies with Pimonidazole in vivo. *Bio Protoc*. 2014;4.
6. Hofer SO, Mitchell GM, Penington AJ, Morrison WA, RomeoMeeuw R, Keramidaris E, Palmer J and Knight KR. The use of pimonidazole to characterise hypoxia in the internal environment of an in vivo tissue engineering chamber. *Br J Plast Surg*. 2005;58:1104-14.
7. Jefferson AL, Cambronero FE, Liu D, Moore EE, Neal JE, Terry JG, Nair S, Pechman KR, Rane S, Davis LT, Gifford KA, Hohman TJ, Bell SP, Wang TJ, Beckman JA and Carr JJ. Higher Aortic Stiffness is Related to Lower Cerebral Blood Flow and Preserved Cerebrovascular Reactivity in Older Adults. *Circulation*. 2018.
